# Plasmid‐mediated horizontal gene mobilisation: Insights from two lactococcal conjugative plasmids

**DOI:** 10.1111/1751-7915.14421

**Published:** 2024-05-16

**Authors:** Guillermo Ortiz Charneco, Brian McDonnell, Philip Kelleher, Andrius Buivydas, Sofia Dashko, Paul P. de Waal, Irma van Rijswijck, Noël N. M. E. van Peij, Jennifer Mahony, Douwe Van Sinderen

**Affiliations:** ^1^ School of Microbiology & APC Microbiome Ireland University College Cork Cork Ireland; ^2^ dsm‐firmenich, Taste, Texture & Health, Center for Food Innovation Delft The Netherlands

## Abstract

The distinct conjugation machineries encoded by plasmids pNP40 and pUC11B represent the most prevalent plasmid transfer systems among lactococcal strains. In the current study, we identified genetic determinants that underpin pNP40‐ and pUC11B‐mediated, high‐frequency mobilisation of other, non‐conjugative plasmids. The mobilisation frequencies of the smaller, non‐conjugative plasmids and the minimal sequences required for their mobilisation were determined, owing to the determination of the *oriT* sequences of both pNP40 and pUC11B, which allowed the identification of similar sequences in some of the non‐conjugative plasmids that were shown to promote their mobilisation. Furthermore, the auxiliary gene *mobC*, two distinct functional homologues of which are present in several plasmids harboured by the pNP40‐ and pUC11B‐carrying host strains, was observed to confer a high‐frequency mobilisation phenotype. These findings provide mechanistic insights into how lactococcal conjugative plasmids achieve conjugation and promote mobilisation of non‐conjugative plasmids. Ultimately, these insights would be harnessed to optimise conjugation and mobilisation strategies for the rapid and predictable development of robust and technologically improved strains.

## Graphical abstract

Lactococcal plasmids pNP40 and pUC11B are shown to display high frequency mobilisation of non‐conjugative smaller plasmids present in the same donor strain. The genetic determinants for this mobilisation are discussed herein.

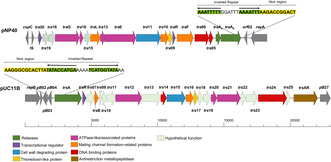

## INTRODUCTION

*Lactococcus lactis* and *Lactococcus cremoris* are widely used in the dairy industry as starter cultures, with *L. cremoris* often associated with slower acidification properties and producing a less bitter end product when compared to *L. lactis* strains (Li et al., 2020). Their widespread and sustained application in food fermentation processes is primarily due to technologically relevant, (predominantly) plasmid‐encoded traits, such as lactose metabolism, casein degradation, bacteriophage resistance and exopolysaccharide production (Fallico et al., 2011).

Several lactococcal plasmids encode conjugation functions which facilitate plasmid transfer from a donor to a recipient cell via a presumed Type IV Secretion System (T4SS) process (Cabezón et al., 2015; Ortiz Charneco et al., 2023). The conjugation‐associated T4SS represents a multiprotein complex that spans the bacterial cell envelope in both Gram‐positive and Gram‐negative bacteria (Grohmann et al., 2018). The mechanism by which conjugation commences is highly conserved among Gram‐positive and Gram‐negative bacteria, as is briefly explained next. The relaxase (or nickase) of the conjugation system introduces a single‐strand (ss) break in the conjugative plasmid at the *nic*‐site located at the origin of transfer (*oriT*), and covalently binds to the 5′‐end of the generated ssDNA, thereby forming the so‐called relaxosome. Subsequently, coupling proteins interact with this relaxosome to facilitate its delivery to the mating channel and then promote its transfer to a recipient cell via an ATP‐dependent translocation mechanism. Simultaneously, plasmid replication ensures that both donor and recipient harbour a double‐stranded (ds) version of the conjugative plasmid (Goessweiner‐Mohr et al., 2014). The main difference between Gram‐negative and Gram‐positive conjugation processes is that the former establishes cell‐to‐cell contact via conjugation pili, whereas the latter appears to rely on cell surface‐associated adhesins (Kohler et al., 2019).

Plasmids that lack a conjugation gene cluster may nonetheless co‐mobilise along with conjugative plasmids, provided they contain a similar *oriT* sequence and at least one mobilisation (*mob*) gene (Francia et al., 2004). These mobilisation genes can be *mobA* or *mobD*, which encode nickases, and *mobB* or *mobC*, encoding auxiliary proteins that constitute the relaxosome along with the associated nickase (O'Brien et al., 2015). It has recently been established that plasmids associated with antibiotic resistance in *Staphylococcus aureus*, though lacking any mobilisation genes to facilitate horizontal transfer, are mobilisable due to the presence of sequences similar to those of the *oriT* of conjugative plasmids (Yui Eto et al., 2021). The relaxase encoded on the conjugative plasmid recognises the *oriT* sequence present in the non‐conjugative plasmid and may thus promote its transfer to a recipient cell (a process referred to as mobilisation). Where both the conjugative and non‐conjugative plasmids are transferred to a recipient cell, the process is termed co‐mobilisation (Francia et al., 2004). The prevalent lactococcal conjugative plasmid pNP40 has previously been shown to mediate co‐mobilisation of two smaller, non‐conjugative plasmids present in the same donor strain at high frequencies (Ortiz Charneco et al., 2021). While the pNP40 conjugation system is the most prevalent system among publicly available lactococcal plasmid sequences, several other lactococcal plasmids harbour homologues of a set of four adjacent, pNP40‐associated conjugation‐related genes, including the *mobD*‐like relaxase and the *mobC*‐like accessory factor (Ortiz Charneco et al., 2021). Despite the absence of a complete set of conjugation‐promoting genes, it was proposed that these plasmids are subject to mobilisation in the presence of the compatible conjugative plasmid pNP40.

The large lactococcal conjugative plasmid pMRC01 facilitates the mobilisation of another plasmid into which the *oriT* sequence from pMRC01 was cloned (Hickey et al., 2001). pMRC01‐like plasmids, among which is the conjugative plasmid pUC11B, form another prevalent conjugation system encoded by certain lactococcal plasmids (Ortiz Charneco et al., 2021). A previous study (Ortiz Charneco et al., 2023) determined that plasmids pMRC01, pAF22 and pUC11B were part of the ‘pMRC01‐like’ group of plasmid‐encoded conjugation systems in lactococci, representing the second most prevalent type of plasmid‐encoded conjugation systems among lactococcal strains. Among these three plasmids, the conjugative plasmid pUC11B remains the least characterised, with pUC11B‐mediated mobilisation not being reported to date. Furthermore, apart from the *oriT*, there is limited knowledge pertaining to the functions of many of the products of the conjugation‐associated genes in the pMRC01 conjugation system (Hickey et al., 2001). Therefore, the pUC11B‐encoded conjugation system is a useful model to extend our understanding of the functions and mechanisms associated with this group of conjugative plasmids, and the presence of a tetracycline‐resistance phenotype associated with this plasmid makes the studies significantly more convenient. Therefore, the current study sought to establish if mobilisation occurs in conjunction with the pUC11B conjugation system and to define the mechanisms underpinning this phenomenon. The findings presented herein provide insights into (a) the frequency/occurrence of mobilisation mediated by pUC11B; (b) the minimal sequences required for mobilisation by pUC11B or pNP40 and (c) genetic determinants that promote a high mobilisation phenotype.

## EXPERIMENTAL PROCEDURES

### Bacterial strains and culture conditions

Bacterial strains used in this study are listed in Table S1. Overnight cultures were prepared by inoculating cells from frozen stocks into 10 mL of M17 supplemented with 0.5% (v/v) glucose (GM17) containing either nisin (2.5 μg/mL, for selection of pNP40), streptomycin (500 μg/mL, to select for the recipient *L. cremoris* MG1614) (Gasson, 1983), tetracycline (10 μg/mL, to select strains harbouring pUC11B or pPTPi) or erythromycin (5 μg/mL, to select for strains containing the mobilisable plasmids, pNZ8048E or pPEPi, or derivatives thereof) (Ortiz Charneco et al., 2023) and incubated at 30°C for 16 h. All plasmids and constructs are summarised in Table S3. Electrocompetent cells of *L. cremoris* were prepared as previously described (Holo & Nes, 1989).

### pUC11B‐mediated plasmid co‐mobilisation

*Lactococcus lactis* UC11 harbours six plasmids named pUC11A to pUC11F (Kelleher et al., 2017). Co‐mobilisation of any plasmid in addition to pUC11B from the donor *L. lactis* UC11 to the recipient *L. cremoris* MG1614 following a solid mating protocol (Ortiz Charneco et al., 2021) was assessed by multiplex polymerase chain reaction (mPCR). This mPCR was performed using seven pairs of primers specific to each of the six plasmids as well as the chromosome of *L. lactis* UC11 (acting as a control to discriminate recipient from donor) (Table S2), each producing differently sized amplicons: UC11 chromosome (1453 bps), pUC11A (1295 bps), pUC11B (1041 bps), pUC11C (816 bps), pUC11D (646 bps), pUC11E (460 bps), pUC11F (322 bps). The mPCR was performed as follows: primers were designed with similar annealing temperatures, and single colonies were randomly selected from the transconjugant plates and added to the mPCR master mix in a total volume of 50 μL and with Phusion Green High‐Fidelity DNA Polymerase (2 U/μL) (Thermo‐Fisher Scientific, Waltham, MA, United States). The conditions for the mPCR included an initial denaturation step at 95°C for 10 min, followed by 35 cycles of 1 min denaturation at 95°C, 30 s annealing at 50°C and 1 min extension at 72°C, followed by a final extension step at 72°C for 7 min. One hundred randomly selected colonies from the transconjugant GM17 agar plates (supplemented with tetracycline and streptomycin) were evaluated using the above‐mentioned mPCR to determine the co‐mobilisation frequency of the different *L. lactis* UC11 plasmids (along with pUC11B) into the recipient *L. cremoris* MG1614. Colonies of the donor and recipient strains were included as controls.

### Plasmids and plasmid constructs

All plasmids and constructs employed in this study are listed in Table S3. Plasmids were purified using the GeneJET Plasmid Maxiprep Kit according to the manufacturer's instructions (Thermo Scientific, USA). Plasmid constructs were assembled using conventional recombinant DNA techniques. PCR fragments used for cloning were digested with the same enzymes as the corresponding cloning vector and ligated using T4 DNA ligase (Promega). Plasmid constructs were introduced by electroporation in *L. cremoris* NZ9000 (Kuipers et al., 1998) and screened for the presence of both plasmid and insert by colony PCR. For the construction of plasmid pNZ8048E, the backbone of pNZ8048 (De Ruyter et al., 1996) without the chloramphenicol resistance gene was amplified using the primers pNZ8048‐Fw/Rv (Table S2), the erythromycin resistance gene from pNZ44E (Draper et al., 2009) was amplified using the primers (Ery‐Fw/Rv); EcoRI and BsrGI digests of these two amplicons were ligated prior to transformation into *Escherichia coli*.

The conjugative plasmids pNP40 and pUC11B were analysed with the oriTfinder software (Li et al., 2018) to identify the potential presence or absence of *oriT*s as a precursor to the cloning of the regions. Plasmids pDRC3E, pDRC3F, pUC11D, pUC11E and pUC11F were amplified as linear fragments by PCR, also incorporating either SacI/SalI, SalI/NcoI or SacI/NcoI recognition sites at the amplicon ends to subsequently ligate to the erythromycin‐resistance (*erm*) gene from pNZ44E (Draper et al., 2009) using T4 DNA ligase (Table S2), thus rendering erythromycin‐resistant derivative versions, with respective nomenclature pDRC3E(e), pDRC3F(e), pUC11D(e), pUC11E(e) and pUC11F(e). To determine the minimal sequences required by these plasmids for (co‐)mobilisation by either pNP40 (pDRC3E or pDRC3F) or pUC11B (pUC11D, pUC11E and pUC11F), successively smaller versions of each mobilisable plasmid were generated by PCR using primers listed in Table S2 and ligated with the erythromycin‐resistance gene. The *mobC* and *mobA* genes of plasmids pDRC3A and pUC11C were cloned individually or in tandem (i.e. *mobC* and *mobA* together, or *mobC* or *mobA* on their own) into the low‐copy number vector pPTPi (O'Driscoll et al., 2004) using the oligonucleotides listed in Table S2. Furthermore, the *oriT*‐containing sequences from either pNP40 or pUC11B were individually cloned into the erythromycin‐resistant derivative pPEPi (Ortiz Charneco et al., 2023), into which the aforementioned *mob* genes from pDRC3A and pUC11C were cloned. All constructs were sequenced by Sanger sequencing (Eurofins, Ebersberg, Germany) to verify sequence integrity.

### Mating and (co‐)mobilisation assays

Conjugation was performed using the spread solid mating approach, as previously described (Ortiz Charneco et al., 2021). Overnight cultures of donor and recipient strains were mixed in a 1:1 volume ratio (representing an approximate 1:1.5 donor/recipient viable cell count ratio), centrifuged at 3000 × *g*, resuspended in 200 μL of 5% reconstituted skim milk (RSM) supplemented with 2% glucose and evenly spread on 5% RSM, 2% glucose agar plates. The plates were incubated overnight at 30°C, after which the cells were scraped from the plates and suspended in 4 mL of Ringer's solution, serially diluted and plated onto GM17 agar plates supplemented with the corresponding antibiotic.

The suspected *oriT*‐containing sequences from plasmids pNP40 or pUC11B were individually cloned in the cloning vector pNZ8048E, resulting in constructs pNZ8048E::*oriT*_pNP40_ and pNZ8048E::*oriT*_pUC11B_, respectively. Furthermore, successively smaller fragments of these regions were cloned individually into pNZ8048E, resulting in constructs pNZ8048E::*oriT*min1‐5_pNP40_ and pNZ8048E::*oriT*min1‐7_pUC11B_, which were introduced into *L. cremoris* NZ9000 pNP40 and *L. cremoris* NZ9000 pUC11B, respectively. The ability of conjugative plasmids pNP40 and pUC11B to co‐mobilise the pNZ8048E vector containing different fragments of their presumed *oriT*‐containing sequences was assessed to determine the minimal sequences required for co‐mobilisation, and thus the possible nick region of either pNP40 or pUC11B.

To select for mobilisation events involving *erm*‐containing derivatives of the native mobilisable plasmids or the pNZ8048E::*oriT* constructs, agar plates were supplemented with 5 μg/mL erythromycin and 500 μg/mL streptomycin (the latter to select for recipient strain *L. cremoris* MG1614). Additionally, determination of co‐mobilisation was assessed using agar plates supplemented with 5 μg/mL erythromycin, 500 μg/mL streptomycin and either 2.5 μg/mL of nisin (for pNP40‐mediated co‐mobilisation) or 10 μg/mL of tetracycline (for pUC11B‐mediated co‐mobilisation). The successful transfer of the mobilisable and/or conjugative plasmid into the recipient cells was further validated by PCR using specific oligonucleotides (Table S2).

To assess the role and impact of *mobA* and *mobC* homologues present in the original donors (*L. lactis* DRC3 or *L. lactis* UC11) on plasmid mobilisation, pPTPi::*mob* constructs were individually introduced into either *L. cremoris* NZ9000 strains harbouring pNP40 or pUC11B and their derivatives harbouring either pDRC3E(e) or pDRC3F(e) in the case of pNP40, or pUC11D(e), pUC11E(e) or pUC11F(e) in the case of pUC11B. To evaluate the effect of induced expression of a given *mob* gene on mobilisation, these strains were individually used as donors with the recipient *L. cremoris* MG1614 and incorporated 10 ng/mL nisin prior to mixing the donor with the recipient strain. The pPEPi constructs harbouring *mob* genes of plasmids pDRC3A and pUC11C, as well as the *oriT*‐containing region from either pNP40 or pUC11B, were further assessed for potential (co‐)mobilisation by either conjugative plasmid by transformation into *L. cremoris* NZ9000 pNP40 or *L. cremoris* NZ9000 pUC11B, followed by conjugation with the recipient strain *L. cremoris* MG1614. Expression of *mob* genes was induced by adding 10 ng/mL nisin. In all cases, conjugation, mobilisation and co‐mobilisation frequencies are expressed as the percentage of the transconjugant colonies (cfu/mL) compared to the recipient colonies (cfu/mL). All assays were performed in triplicate.

### Statistical data analysis

Data in this study represent the means ± standard deviation (SD) of triplicate assays. Results were analysed using the SigmaPlot 11.0 statistical package (SPSS), from Systat Software, Inc., San Jose, California, USA. A *p* value of <0.05 was considered significant and is represented in the graphs with a single asterisk ‘*’, while a *p* value of ≤0.001 was considered very significant and is represented by two asterisks ‘**’ in the graphs.

## RESULTS

### pUC11B facilitates co‐mobilisation of plasmids

Plasmid mobilisation mediated by lactococcal conjugative plasmids represents a potential tool for the improvement of starter cultures applied in the dairy fermentation industry. Prior studies in the streptococcal conjugative plasmid pIP501 have shown that the location of the *mob* genes may be a hot spot for cointegrate formation and promote mobilisation of non‐conjugative plasmids (Langella et al., 1993). Additionally, it has been reported that the presence of a similar *oriT* sequence in a non‐conjugative vector to that of the conjugative plasmid pMRC01 promotes the mobilisation of the vector during conjugation with pMRC01 (Hickey et al., 2001). However, there is still scarce information regarding the mobilisation of naturally occurring non‐conjugative plasmids in *Lactococcus* when these are in the same donor strain as conjugative plasmids. Furthermore, it remains uncertain whether in lactococcal species the presence of *mob* genes and *oriT*‐like sequence in a non‐conjugative plasmid suppose an added factor for an increase in mobilisation frequency, a subject that has been studied in the Gram‐negative model *E. coli* (Garcillán‐Barcia et al., 2019). Thus, the study of the genetic determinants that promote mobilisation of non‐conjugative plasmids in *Lactococcus* remains a compelling subject of study.

**FIGURE 1:**
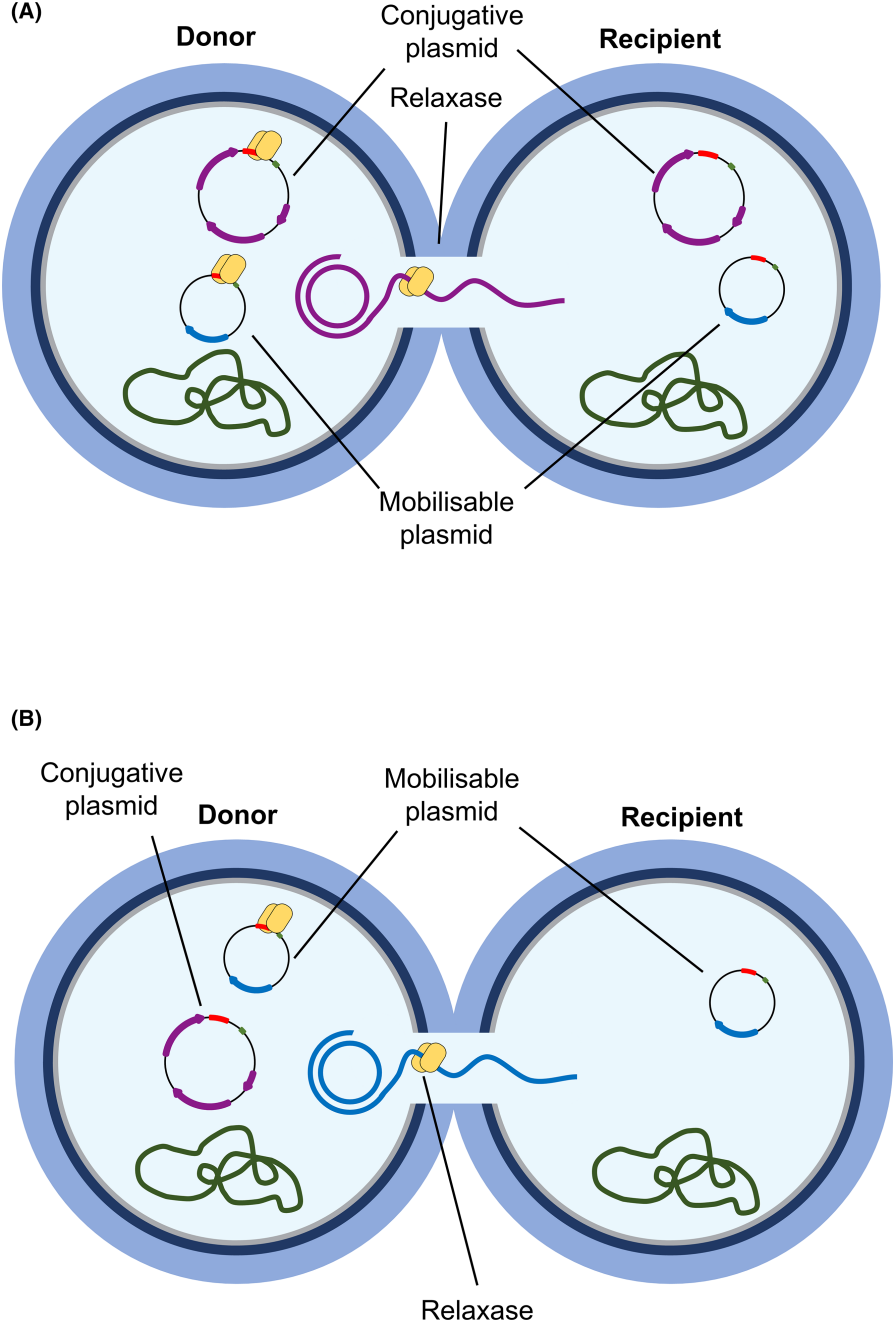
Schematic representation of conjugation‐mediated plasmid (co‐)mobilisation between two Gram‐positive bacteria. (A) Plasmid co‐mobilisation mediated by the transfer of a conjugative plasmid. The relaxase encoded by the conjugative plasmid initiates the transfer of this plasmid by introducing a ssDNA break. This relaxase would, presumably, recognise a similar sequence in other smaller and non‐conjugative plasmid present in the same donor strain, thus also promoting the transfer of said plasmid. Once the conjugation event is finalised, the recipient cell will end up with a copy of the conjugative plasmid and the smaller, non‐conjugative plasmid. (B) Plasmid mobilisation. This phenomenon not only encompasses the events of plasmid co‐mobilisation, those in which both the conjugative and non‐conjugative plasmids are transferred into the same recipient cell, but also those events in which the relaxase from the conjugative plasmid promotes the transfer of the smaller, non‐conjugative plasmid into the recipient cell, but does not promote the transfer of the conjugative plasmid itself.

Plasmid co‐mobilisation has been reported for lactococcal plasmids pDRC3E and pDRC3F and is mediated by conjugative plasmid pNP40 (Ortiz Charneco et al., 2021). Plasmid mobilisation refers to those events wherein non‐conjugative plasmids are transferred into a recipient cell after conjugation, in the presence or absence of the conjugative plasmid, whereas co‐mobilisation refers to the events in which both conjugative and non‐conjugative plasmids are transferred from the donor to recipient cell after conjugation (Figure 1).

To establish the extent of this horizontal gene transfer phenomenon among (other) lactococcal conjugative systems, the pUC11B‐encoded conjugative system was evaluated for its ability to mediate plasmid co‐mobilisation. *L. lactis* UC11 harbours six plasmids: pUC11A (59,284 kb), pUC11B (49,307 kb), pUC11C (19,351 kb), pUC11D (15,393 kb), pUC11E (7809 kb) and pUC11F (5238 kb) (Kelleher et al., 2017). A multiplex PCR (or mPCR) approach was designed to assess possible plasmid transfer events from the donor strain to a recipient and incorporated seven sets of primers, each targeting unique sequences within each of the six plasmids (and a chromosomal control) from *L. lactis* UC11.

**FIGURE 2:**
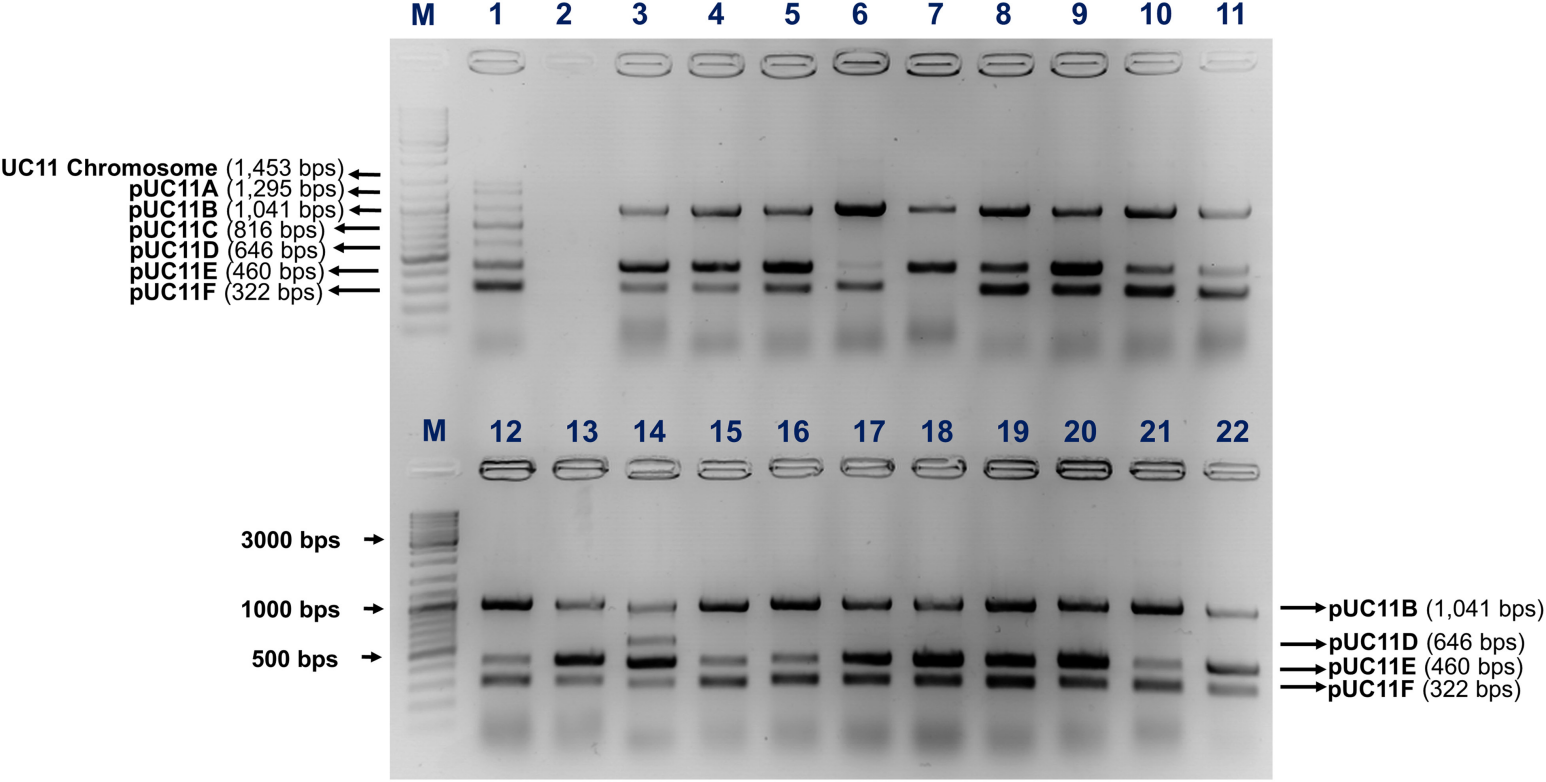
Agarose gel displaying the mPCR (involving seven primer pairs) profiles of 20 representative transconjugants. Lane M corresponds to the DNA ladder used (GeneRuler DNA Ladder Mix). Lane 1 corresponds to the positive control, the donor strain *L. lactis* UC11, in which a product for each primer pair can be observed, whereas lane 2 is the negative control, the recipient strain *L. cremoris* MG1614, in which no product is observed. The remaining lanes (3–22) represent 20 randomly selected transconjugants. Plasmid pUC11B was transferred to all colonies as expected due to selection with tetracycline. Plasmid pUC11E was also transferred to all analysed colonies while pUC11F and pUC11D were co‐mobilised in 94% and 7% of the transconjugants screened, respectively.

Employing a solid mating protocol (see Experimental Procedures), conjugation assays were performed between *L. lactis* UC11 (donor) and *L. cremoris* MG1614 (recipient), achieving a conjugation frequency of 1.14%, and 100 transconjugant colonies were randomly selected for mPCR screening of co‐mobilisation events. As expected, pUC11B was present in all screened colonies confirming the successful conjugation event (Figure 2; this figure shows the PCR results for 20 of the 100 randomly selected transconjugants). Additionally, the two smallest plasmids, pUC11E and pUC11F, were shown to be co‐mobilised by pUC11B in 100% and 94% of the tested transconjugant colonies, respectively, while pUC11D was present in 7% of the screened transconjugants. Interestingly, in one case we showed that four plasmids had been transferred to a single recipient cell (Figure 2). Out of the 100 random transconjugant colonies screened, mobilisation of the smaller plasmids always occurred alongside the transfer of the conjugative plasmid pUC11B.

### pUC11B and pNP40 mediate (co‐)mobilisation of plasmids containing their native *oriT*

The observed high‐frequency co‐mobilisation of plasmids during pUC11B‐ and pNP40‐mediated conjugation (Ortiz Charneco et al., 2021), may be due to the presence of *oriT*‐like sequences in the co‐mobilised plasmids as has been suggested previously for the conjugative plasmid pMRC01 (Hickey et al., 2001). To identify the *oriT* sequence and possible nick region of either pNP40 or pUC11B, the region upstream of *trsA* in pUC11B, and the region downstream of *traA*_*b*_ in pNP40, were individually cloned into pNZ8048E. These regions were selected based on the premise that an *oriT* sequence is typically located in an intergenic region close to the gene encoding its cognate DNA relaxase (Lee & Grossman, 2007). Furthermore, although the web‐based software oriTfinder (Li et al., 2018) did not predict any *oriT* regions in neither pNP40 nor pUC11B, it showed that the aforementioned sequences were potential candidates for harbouring the presumed *oriT* regions of their respective conjugative plasmids.

**FIGURE 3:**
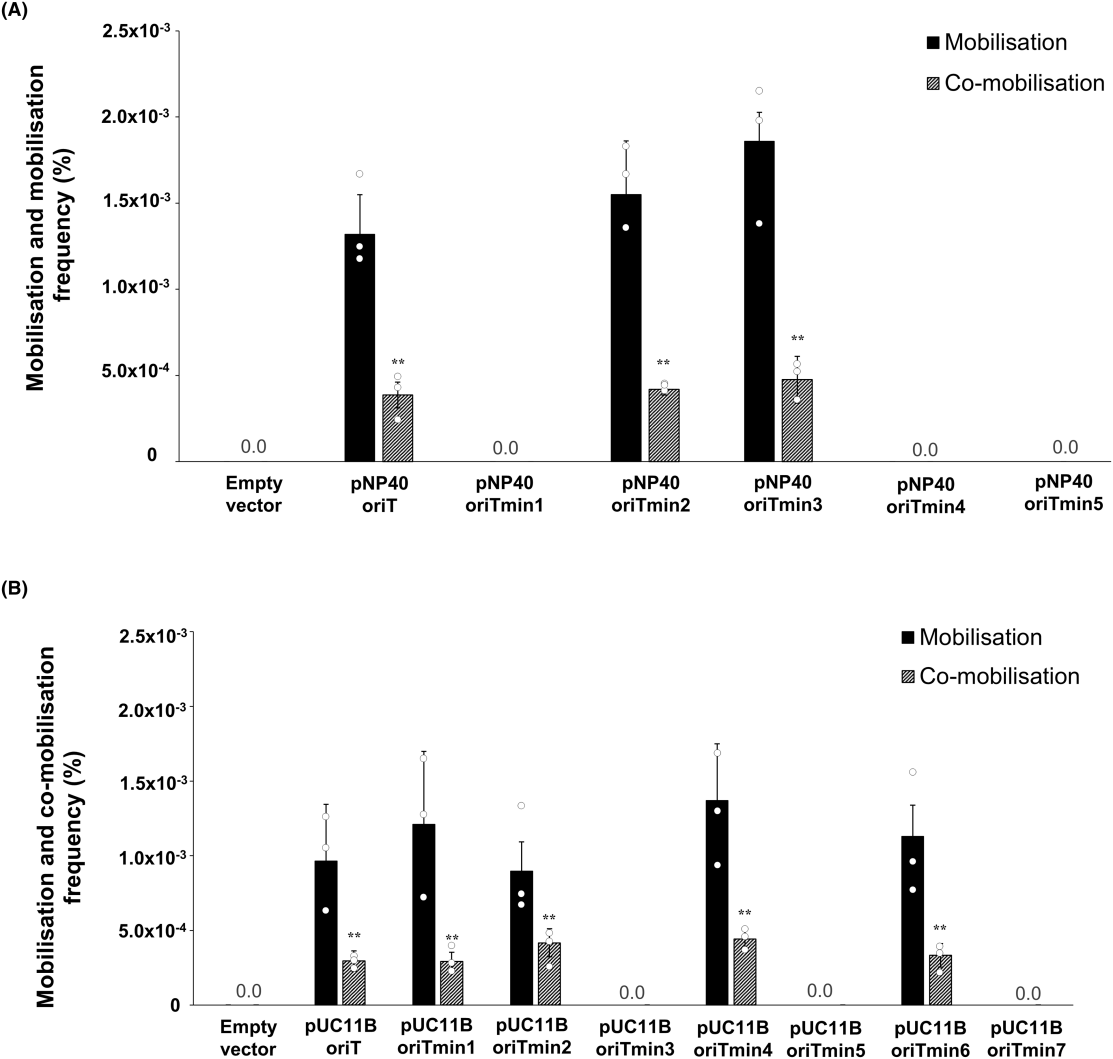
(A) (Co‐)mobilisation frequencies of the pNZ8048E constructs harbouring the different fragments from the presumed oriT sequence of plasmid pNP40; (B) (Co‐)mobilisation frequencies of the pNZ8048E constructs harbouring the different fragments from the presumed oriT sequence of plasmid pUC11B. In all cases, co‐mobilisation frequencies are compared to mobilisation frequencies, being the mobilisation frequencies in all the instances in which only the corresponding pNZ8048E construct was tracked, and the comobilisation frequencies in those events in which both the transfer of the corresponding pNZ8048E construct and the conjugative plasmid (either pNP40 or pUC11B) were successful. A *p*‐value ≤0.001 was considered very significant and is represented by two asterisks ‘**’. Presented data are the mean of three replicates ± standard deviation. Constructs that displayed no (co‐)mobilisation frequencies refer to frequencies below our limit of detection (<1 × 10^−5^%).

Our results showed that pNZ8048E constructs containing the presumed *oriT* sequences from either pNP40 or pUC11B were (co‐)mobilised by the respective conjugative plasmid during conjugation (Figure 3), with the conjugative plasmids themselves being self‐transferred at previously reported frequencies (Ortiz Charneco et al., 2021, 2023). The empty pNZ8048E plasmid control did not exhibit detectable (co‐)mobilisation (limit of detection <1 × 10^−5^%) in either of the two test systems. The presence of an antibiotic resistance marker in pNZ8048E allows discrimination between plasmid mobilisation and co‐mobilisation, and we found that the frequencies of mobilisation were significantly (*p* ≤ 0.001) higher than the co‐mobilisation frequencies. Successively smaller versions of these presumed *oriT*‐containing regions were generated by PCR and cloned into pNZ8048E to establish the minimal sequences present within these regions required for plasmid (co‐)mobilisation (further details presented in Table S4). Five pNZ8048E, pNP40‐based *oriT*‐containing constructs of different sizes (Figure 3A) were individually introduced into *L. cremoris* NZ9000 pNP40, and seven pNZ8048E constructs containing various fragments from the intergenic region upstream of the relaxase‐encoding gene *trsA* from pUC11B were similarly introduced into *L. cremoris* NZ9000 pUC11B and the resulting strains were then used as donors against *L. cremoris* MG1614. Among the five pNP40‐based smaller constructs (Figure 3A), two were co‐mobilised by pNP40, at a frequency of ≃4 × 10^−4^%, suggesting that the presence of the pNP40 *oriT* sequence facilitated their co‐mobilisation. The other three constructs did not facilitate (co‐)mobilisation within the limit of detection of the assay <1 × 10^−5^%, suggesting that an essential part of the presumed *oriT* required for mobilisation was absent in these plasmids. Similarly, four out of the seven smaller pUC11B‐based constructs were shown to be co‐mobilised by pUC11B (Figure 3B), at co‐mobilisation frequencies of ≃4 × 10^−4^%, and thus it was assumed that these constructs contain the *oriT* of plasmid pUC11B.

Interestingly, no (co‐)mobilisation was observed (limit of detection <1 × 10^−5^%) for the pNZ8048E construct containing the presumed *oriT* from pUC11B when it was introduced into *L. cremoris* NZ9000 pNP40 and vice versa, showing that the encoded conjugation machinery from either pNP40 or pUC11B only recognises their specific *oriT* sequences, and is apparently incapable of (co‐)mobilising plasmid DNA containing a non‐homologous *oriT* sequence originating from another conjugative plasmid (data not shown).

**FIGURE 4:**
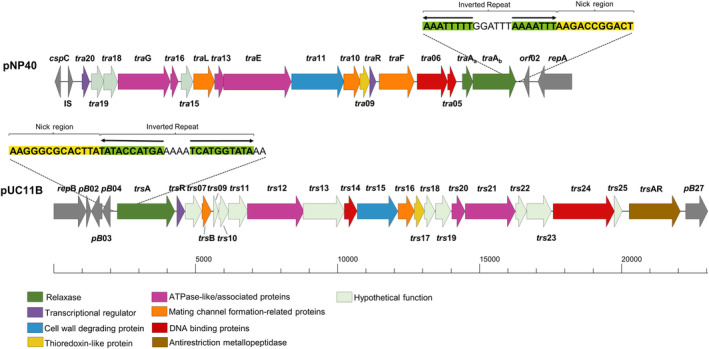
Schematic representation of the pNP40 and pUC11B conjugation gene clusters, displaying a colour‐coded representation of the shared protein functionalities between the clusters, based on mutational and predictive functional analysis based on sequence and structural similarities, adapted from Ortiz Charneco et al. (2021, 2023). The oriT regions from both plasmids is highlighted, with the IRs highlighted in green and the presumed nick region in yellow.

Investigated *oriT* regions from conjugative plasmids all contain a conserved nick region flanking the *nic*‐site, which is the precise point of recognition and cleavage of the plasmid‐encoded relaxase, and a variable number of inverted repeats (IRs), which are also involved in relaxase binding and termination of ssDNA transfer (Furuya & Komano, 2000; Li et al., 2018). Plasmids pNP40 and pUC11B were analysed for *oriT* sequences by using the oriTfinder software (Li et al., 2018), followed by the experimental search and validation. The total number of active constructs from the presumed *oriT* region of plasmid pNP40 was 3, whereas 5 out of the 8 constructs tested from the presumed *oriT* region of plasmid pUC11B did (co‐)mobilise. A Clustal Omega alignment (Procter et al., 2021) of the fragments containing the presumed *oriT* sequences from either pNP40 (Figure S1A) or pUC11B (Figure S1B) revealed the minimal sequences required to be present for the (co‐)mobilisation of the pNZ8048E constructs. These minimal *oriT* sequences had a size of 32 bases (including an IR with a palindrome length of 8 bases) and 39 bases (with an IR with a palindrome length of 10 bases) for plasmids pNP40 and pUC11B, respectively (Figure 4A–C). A nick region was identified in both cases based on its adjacency to the IR, which has the potential to generate a hairpin‐like structure that would allow specific recognition of the presumed *oriT* region by the cognate DNA relaxase (Grohmann et al., 2003). Validation of the importance of these sequences in mobilisation will require further deletion and mutational analyses.

### Minimal sequences required for plasmid (co‐)mobilisation reveal similar *oriT* regions

Following the identification of the presumed *oriT* and nick sequences of pNP40 and pUC11B, the presence and role of related sequences in mobilisable plasmids during the mobilisation process was investigated. Erythromycin‐resistant derivatives of the mobilisable plasmids present in the pNP40 (pDRC3E and pDRC3F) and the pUC11B background (pUC11D, pUC11E and pUC11F) were generated (nomenclature (e), see Experimental Procedures), to reliably assess and determine their (co‐)mobilisation frequencies. Furthermore, successively smaller versions of these (erythromycin cassette‐containing) plasmids were generated. All constructs were introduced into either *L. cremoris* NZ9000 pNP40 or *L. cremoris* NZ9000 pUC11B, and the resulting strains were used as donors using a spread solid mating approach with the recipient strain *L. cremoris* MG1614. In all of these conjugation experiments, the obtained conjugation frequencies of pNP40 and pUC11B were similar to those previously reported (Table S5) (Ortiz Charneco et al., 2021, 2023).

**FIGURE 5:**
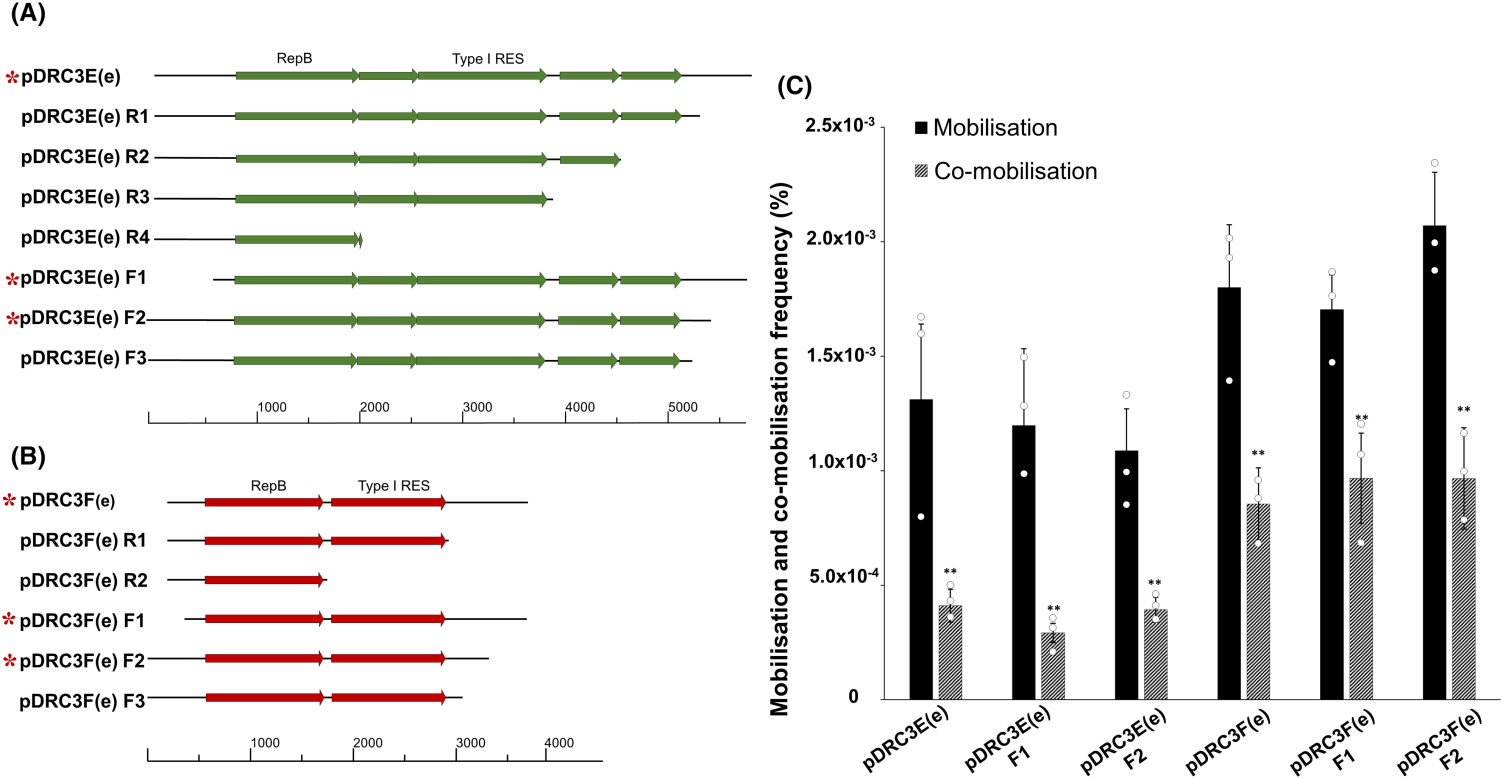
Portions of plasmids pDRC3E (A) and pDRC3F (B), respectively, used to determine mobilisation‐associated sequences. These plasmid sections were ligated to an erythromycin resistance gene from pNZ44E (Draper et al., 2009), and the resulting constructs were named pDRC3E(E)F1‐3 or R1‐4 and pDRC3F(e)F1‐3 or R1‐2. All constructs were introduced into and shown to be able to replicate in *L. cremoris* NZ9000 pNP40. A red asterisk next to the name indicates that (co‐)mobilisation was detectable; (C) Co‐mobilisation and mobilisation frequencies of the pDRC3E(e) and pDRC3F(e) constructs, after conjugation using *L. cremoris* NZ9000 pNP40 harbouring the different constructs, and *L. cremoris* MG1614 as the recipient. Comobilisation frequencies were compared to mobilisation frequencies, and a *p*‐value ≤0.001 was considered very significant and is represented by two asterisks ‘**’. Presented data are the mean of three replicates ± standard deviation. Specific details concerning each plasmid construct can be found in Table S5. Constructs that displayed no (co‐)mobilisation frequencies refer to frequencies below our limit of detection (<1 × 10^−5^%).

Successively smaller sections of these non‐conjugative plasmids were selected based on including or excluding intergenic regions within each plasmid. This initial criteria selection was undertaken under the presumption that an *oriT*‐like sequence may be present in an intergenic region. However, after initial (co‐)mobilisation studies, additional non‐conjugative plasmid derivatives were constructed to try and determine the minimal sequences required for their (co‐)mobilisation. Eight derivatives of plasmid pDRC3E(e) encompassing successively smaller sections of this plasmid were created (Figure 5A), while six derivatives of plasmid pDRC3F(e) were constructed in the same manner (Figure 5B). Each of the constructs was individually introduced into *L. cremoris* NZ9000 pNP40 and subsequently used as donors against *L. cremoris* MG1614, and the mobilisation and co‐mobilisation abilities of the various pDRC3E(e) and pDRC3F(e) constructs assessed. To note, no mobilisation or co‐mobilisation was observed when these constructs were introduced into *L. cremoris* NZ9000 pUC11B (data not shown). When mobilisation occurred, the obtained mobilisation frequencies ranged from 1.09 × 10^−3^% to 2.07 × 10^−3^% and were significantly (*p* ≤ 0.001) higher than the corresponding co‐mobilisation frequencies, which ranged from 2.92 × 10^−4^% to 9.67 × 10^−4^% (Figure 5C). The derivatives that displayed (co‐)mobilisation frequencies lower than our limit of detection (<1 × 10^−5^%) were presumed to be incapable of (co‐)mobilisation (not displayed in Figure 5C).

Using the same approach, seven derivatives of plasmid pUC11D (Figure S2A), nine derivatives of pUC11E (Figure S2B) and six derivatives of pUC11F (Figure S2C) were constructed. Resulting constructs were individually introduced into *L. cremoris* NZ9000 pUC11B and used as donors with the recipient *L. cremoris* MG1614, and the mobilisation and co‐mobilisation frequencies of the constructs were determined. As per the previous experiment, no mobilisation or co‐mobilisation was observed when these constructs were introduced into *L. cremoris* NZ9000 pNP40 (data not shown). Conjugation frequencies of pUC11B were similar to those previously reported using this same plasmid (Ortiz Charneco et al., 2023). Eleven of the 22 constructs were observed to (co‐)mobilise (Figure S2D) with a detection limit of <1 × 10^−5^%. Similar to the pDRC3E(e) and pDRC3F(e) constructs, the pUC11D(e), pUC11E(e) and pUC11F(e) constructs that did mobilise displayed significantly (*p* ≤ 0.001) higher levels of mobilisation (ranging from 5.51 × 10^−4^% to 8.46 × 10^−4^%) than co‐mobilisation (varying from 1.77 × 10^−4^% to 2.62 × 10^−4^%).

Comparison of the minimal sequences that plasmids pUC11D(e), pUC11E(e) and pUC11F(e) required for mobilisation with the *oriT* sequence of the conjugative plasmid pUC11B revealed some interesting similarities (Figure S3). In the case of plasmids pUC11D(e) and pUC11F(e), these minimal mobilisation sequences were 173 and 160 bps long, respectively, and both contained a region of thirteen nucleotides (highlighted in yellow in Figure S3), each displaying just four nucleotide mismatches when compared to the thirteen nucleotides of the pUC11B nick region, which is the region containing the as of yet undetermined *nic*‐site. These minimal sequences also contained IRs relatively close to the nick‐resembling regions. Nevertheless, the highest similarity was that displayed by the minimal sequence required for the mobilisation of plasmid pUC11E(e) (195 bps). This minimal sequence contained an identical nick‐region and IRs to those present in pUC11B, suggesting that these sequences are responsible for pUC11B‐mediated mobilisation. It is noteworthy that plasmid pUC11A harbours the same sequence as pUC11E, including the nick region and IRs of plasmid pUC11B, although this large plasmid (59.284 kb) was not observed to mobilise under the tested conditions. Remarkably, the minimal sequences required for mobilisation of plasmids pDRC3E(e) (122 bps) and pDRC3F(e) (189 bps) did not display any significant similarities with the *oriT* sequence from the conjugative plasmid pNP40, and the genetic elements required for the observed mobilisation of these plasmids remains to be established.

### System‐specific mobilisation genes promote a high co‐mobilisation phenotype

**FIGURE 6:**
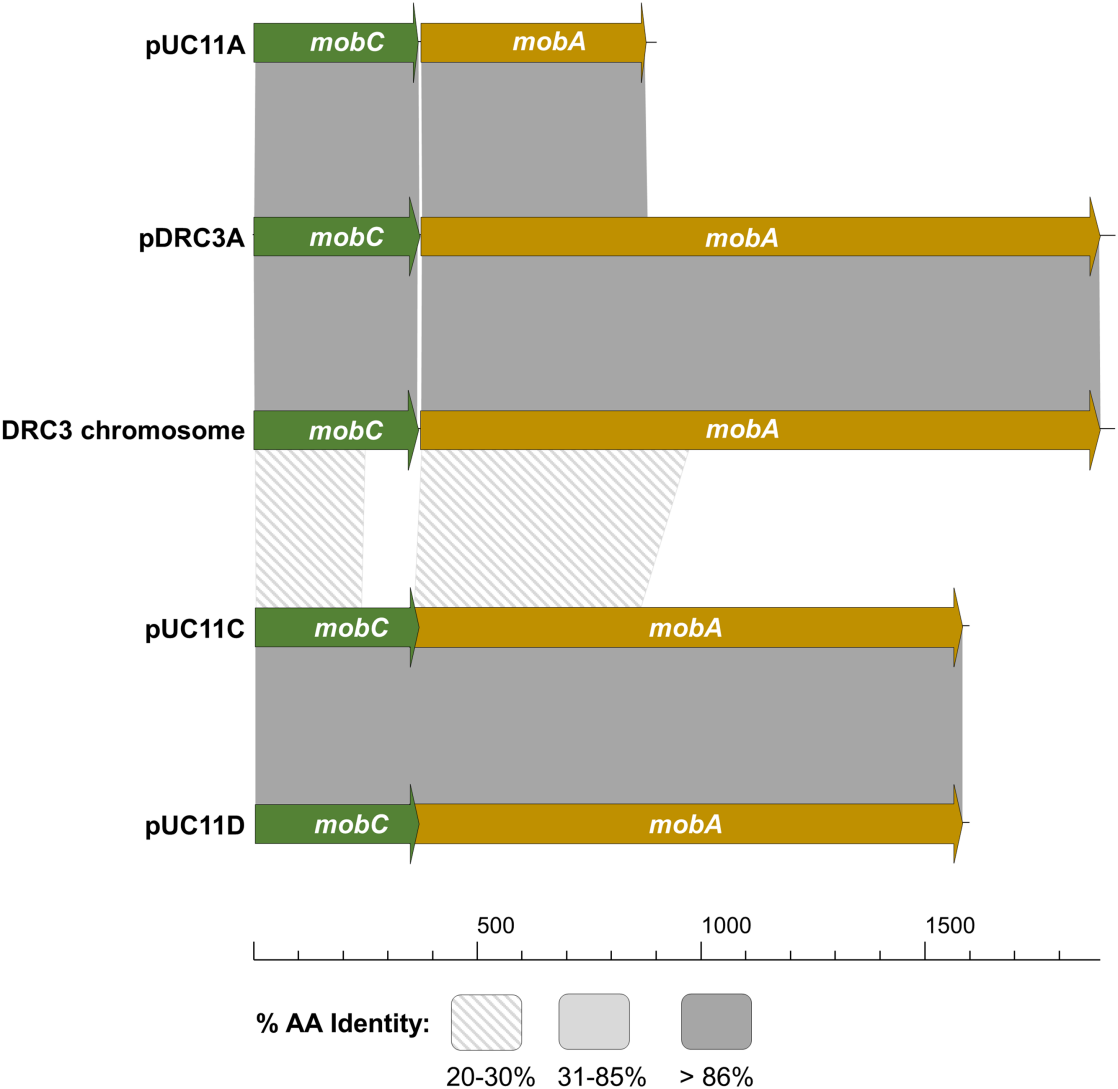
Gene map of the mobC and mobA genes from plasmids pDRC3B (pNP40, associated with its conjugation gene cluster), pUC11A, DRC3 chromosome, pDRC3A, pUC11C and pUC11D. The image shows their amino acid identity, which is indicated by the shaded boxes.

Co‐mobilisation frequencies of plasmids pDRC3E(e), pDR3F(e), pUC11D(e), pUC11E(e) and pUC11F(e), as well as those of the pNZ8048E constructs harbouring the *oriT* sequences from either pNP40 or pUC11B ranged from 1.77 × 10^−4^% to 1.09 × 10^−3^%, representing significantly lower frequencies than those observed in the original co‐mobilisation screens for pDRC3E (≃30%) and pDRC3F (≃99%) (Ortiz Charneco et al., 2021), and those of pUC11D (≃7%), pUC11E (≃100%) and pUC11F (≃94%) evaluated in the present study. In all these experiments, conjugation frequencies of the conjugative plasmids pNP40 and pUC11B were similar to those previously reported (Ortiz Charneco et al., 2021, 2023). Co‐mobilisation frequencies of the derivatives harbouring the *erm* gene were assessed using either *L. cremoris* NZ9000 pNP40 or *L. cremoris* NZ9000 pUC11B as donors, which are not the original hosts of these conjugative plasmids. This apparent incongruity suggests that additional element(s) present in the original donors promote the observed high‐frequency co‐mobilisation. To evaluate this hypothesis, the genomic content of both strains was analysed, and several plasmid‐borne *mob* genes were identified as potential catalysts of the high co‐mobilisation phenotype. Among the seven and six plasmids harboured by *L. lactis* DRC3 and UC11, respectively, pUC11A, pUC11C, pUC11D, pDRC3A and the chromosome of DRC3 harbour a set of *mob* genes (Figure 6). To note, the conjugative plasmid pNP40 harbours a very similar set of mobilisation‐related genes, *traA*_*a*_ (*mobC*) and *traA*_*b*_ (*mobA*).

**FIGURE 7:**
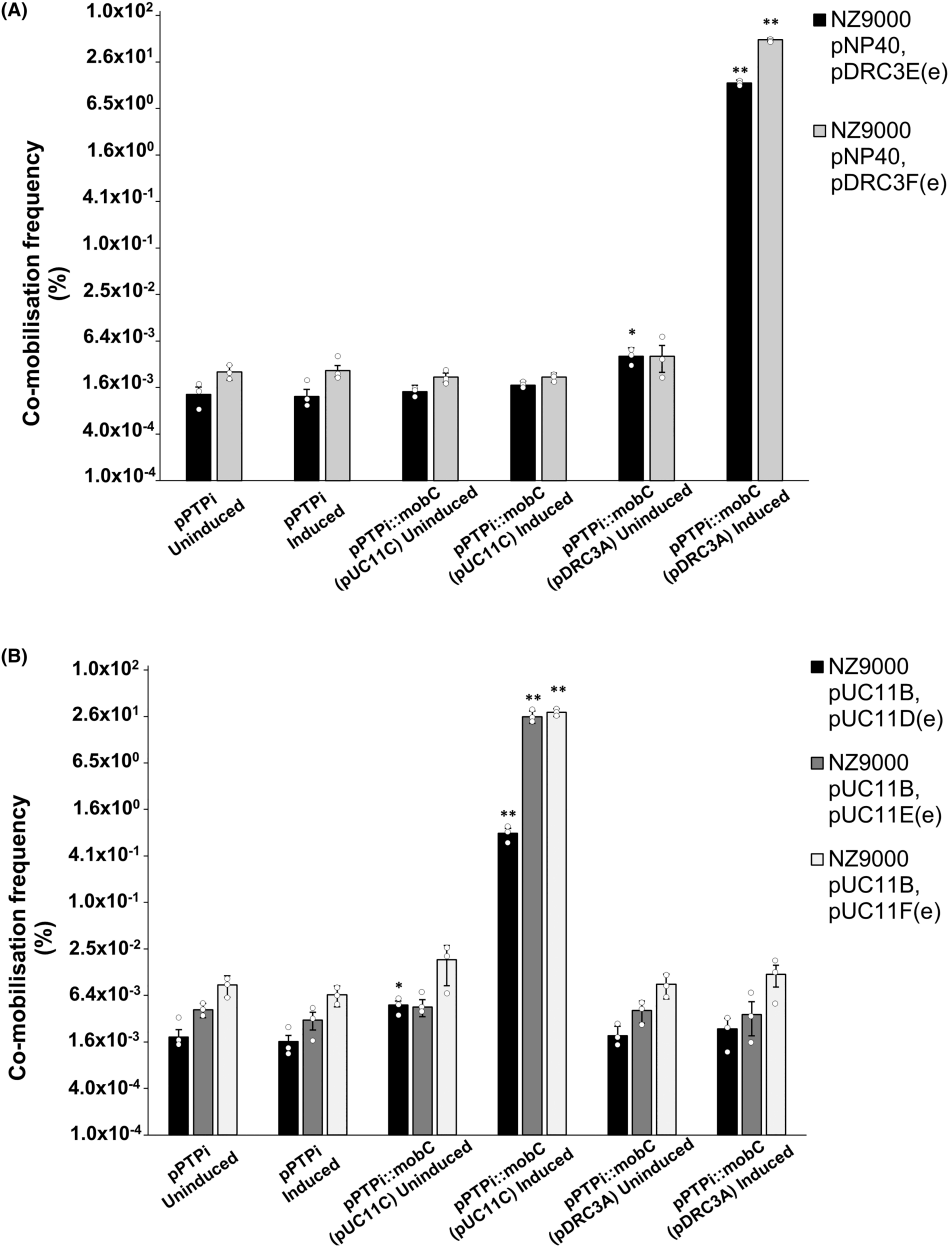
(A) Co‐mobilisation frequencies using either *L. cremoris* NZ9000 pNP40, pDRC3E(e) or pDRC3F(e) as donors against the recipient *L. cremoris* MG1614. (B) Co‐mobilisation frequencies using either *L. cremoris* NZ9000 pUC11B, pUC11D(e), pUC11E(e) or pUC11F(e) as donors against the recipient *L. cremoris* MG1614. In both cases, marked in the x‐axis are the pPTPi constructs that were individually introduced into each donor strain prior to the conjugation event, and the effect that each construct had in the co‐mobilisation frequencies of the erythromycin‐resistant plasmid derivatives can be observed. Complete expression of the mob genes was achieved by adding 10 ng/mL nisin to the growth medium (induced), whereas uninduced mob genes were not subjected to the presence of nisin. Co‐mobilisation frequencies were compared to those obtained with the control (empty pPTPi, uninduced). A *p*‐value <0.05 was considered significant and is represented by a single asterisk ‘*’, whereas a *p*‐value ≤0.001 was considered very significant and is represented by two asterisks ‘**’. Presented data are the mean of three replicates ± standard deviation.

The MobC proteins encoded by the genes located in pUC11A, pNP40, pDRC3A and the *L. lactis* DRC3 chromosome exhibit high amino acid identity among each other (>86%), while those encoded by pUC11C and pUC11D are significantly different (<24% amino acid identity), while being identical between them. Consequently, the *mobC* genes from plasmids pUC11C and pDRC3A were cloned under the control of a nisin‐inducible promoter, and the resulting constructs were introduced into *L. cremoris* NZ9000 strains harbouring pNP40 and either pDRC3E(e) or pDRC3F(e), as well as into *L. cremoris* NZ9000 pUC11B harbouring pUC11D(e), pUC11E(e) or pUC11F(e). When these strains were used as donors in combination with the recipient *L. cremoris* MG1614, no significant differences in the conjugation frequency of neither pNP40 nor pUC11B were observed, irrespective of nisin‐mediated induction. These donor strains were then used together with the recipient *L. cremoris* MG1614 to determine if and how co‐mobilisation of the erythromycin‐resistant derivatives of the mobilisable plasmids was affected (Figure 7).

Nisin‐mediated induction of the *mobC* gene from pDRC3A in a donor strain harbouring the pDRC3E(e) or pDRC3F(e) derivatives was shown to significantly increase the co‐mobilisation frequency of these plasmids to 14% and 51%, respectively, representing an approximate 20,000‐fold increase relative to their co‐mobilisation frequencies in the absence of pDRC3A‐encoded MobC (Figure 7A). Nisin‐mediated induction of *mobC* transcription was shown to be required for the full increase of co‐mobilisation frequency, although in the absence of nisin‐induction co‐mobilisation of pDRC3E(e) was observed to be significantly (*p* < 0.05) increased 2.5‐fold. This increase is presumed to be due to the “leaky” nature of the nisin promoter. Remarkably, nisin‐induced expression of *mobC* from pUC11C did not cause any discernible effect in the co‐mobilisation frequency of the pDRC3E(e) or pDRC3F(e) plasmids, suggesting that the MobC proteins act in an *oriT*‐specific manner. Nisin‐mediated induction of *mobC* from pDRC3A in *L. cremoris* NZ9000 pUC11B harbouring pUC11D(e), pUC11E(e) or pUC11F(e) had no discernible effect on their co‐mobilisation. However, nisin‐mediated induction of the *mobC* gene from pUC11C in *L. cremoris* NZ9000 pUC11B pUC11D(e), pUC11E(e) and pUC11F(e) increased their co‐mobilisation frequency to 0.8%, 26% and 30%, respectively, representing an approximate 400‐, 6000‐ and 3300‐fold increase in their co‐mobilisation frequencies compared to their respective control (Figure 7B). Also in this case, it was obvious that nisin induction of the *mobC* genes is required to observe this effect, although uninduced *mobC* from pUC11C had a significant (*p* < 0.05) effect in the co‐mobilisation pUC11D(e), presumably due to the “leaky” nature of the nisin promoter.

In all the above‐mentioned cases, additional assays were performed in which the *mobA* genes from pDRC3A and pUC11C were cloned individually (data not shown), which yielded no difference in the co‐mobilisation of the erythromycin derivative plasmids when compared to the empty vector. Furthermore, the *mobC* genes were also cloned in tandem with their associated *mobA* genes (data not shown). Co‐expression of both genes did not increase/alter the co‐mobilisation frequency of any of the erythromycin‐derivative plasmids compared to individual *mobC* expression. These findings suggest that the *mobC* genes alone are responsible for the enhanced co‐mobilisation phenotype under the conditions tested.

### *mobC* genes and *oriT* sequences are key components to promote high‐frequency (co‐)mobilisation

It was determined that the presence of a cognate *oriT* sequence from either pNP40 or pUC11B in a plasmid promoted (co‐)mobilisation of this plasmid by the corresponding conjugative plasmid. Furthermore, nisin‐induced expression of a corresponding *mobC* gene significantly increased co‐mobilisation of the erythromycin derivatives of the mobilisable plasmids pDRC3E(e), pDRC3F(e), pUC11D(e), pUC11E(e) and pUC11F(e), respectively. To further verify the combined role that naturally occurring *oriT* sequences and *mobC* gene combinations play during (co‐)mobilisation, the *oriT*‐containing regions from either pNP40 or pUC11B were individually cloned into pPEPi together with their corresponding *mob* genes (from pDRC3A or pUC11C, respectively). The resulting constructs were subsequently introduced into *L. cremoris* NZ9000 harbouring either pNP40 or pUC11B, and the obtained strains were used as donors with *L. cremoris* MG1614 as the recipient.

**FIGURE 8:**
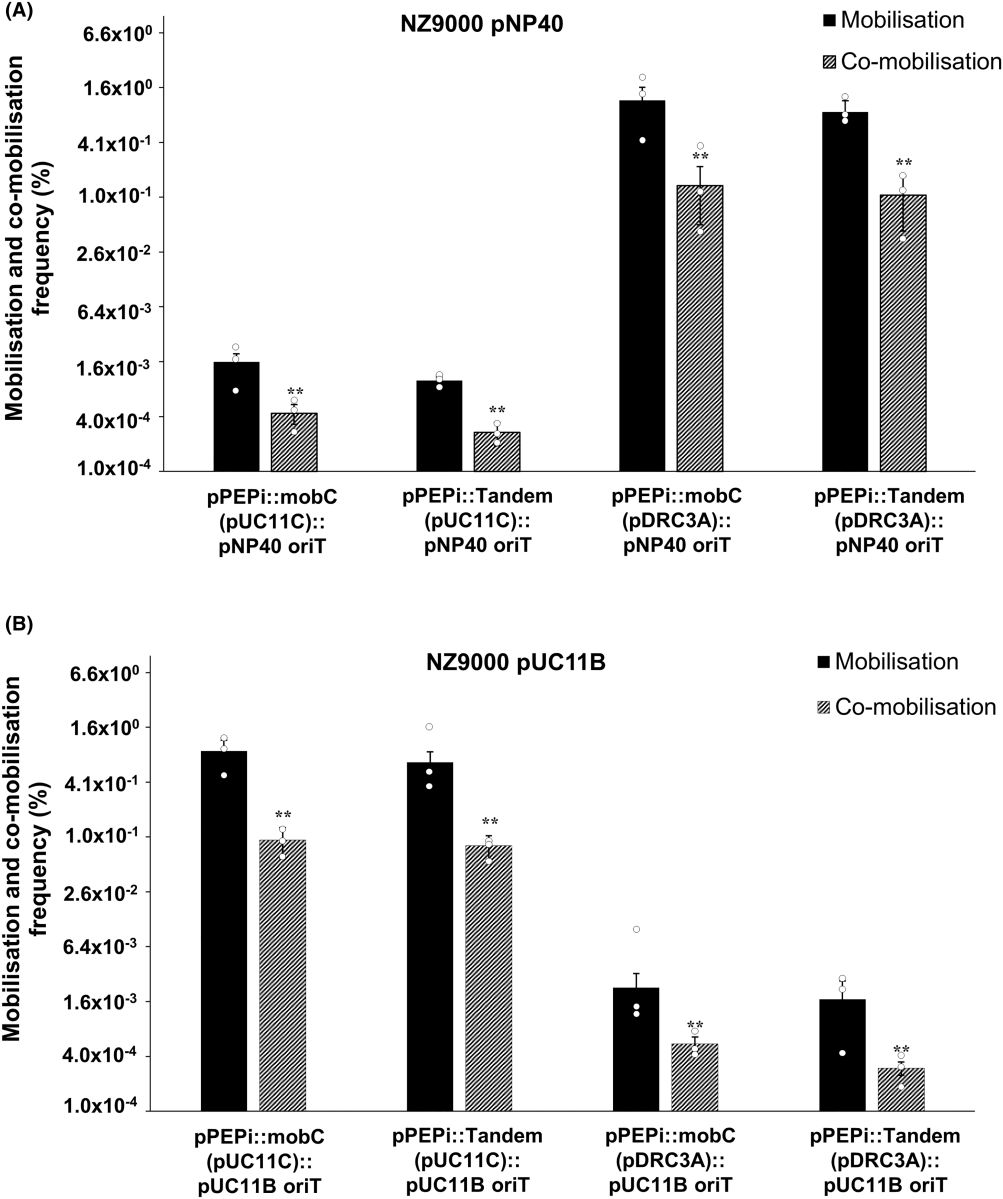
(A) (Co‐)mobilisation frequencies of the pPEPi constructs harbouring the mobC genes from either pUC11C or pDRC3A, as well as the oriT region from pNP40, in the presence of the conjugative plasmid pNP40. (B) (Co‐)mobilisation frequencies of the pPEPi constructs harbouring the mobC genes from either pUC11C or pDRC3A, as well as the oriT region from pUC11B, in the presence of the conjugative plasmid pUC11B. In all cases, the cloned mobC genes were either induced by adding 10 ng/mL of nisin to the growth medium or uninduced (in the absence of nisin). Co‐mobilisation frequencies are compared to mobilisation frequencies, and a *p*‐value ≤0.001 was considered very significant and is represented by two asterisks ‘**’. Presented data are the mean of three replicates ± standard deviation.

Following conjugation, (co‐)mobilisation of the pPEPi constructs was assessed (Figure 8). (Co‐)mobilisation by either pNP40 (Figure 8A) or pUC11B (Figure 8B) occurred where the cognate *oriT* was cloned. Remarkably, and in consonance with results presented above (Figure 7), (co‐)mobilisation frequencies of the pPEPi constructs by both pNP40 and pUC11B were significantly (*p* ≤ 0.001) higher when the corresponding *mobC* genes (from pDRC3A or pUC11C, respectively) were expressed via nisin‐mediated induction, with ≃1 × 10^−1^% co‐mobilisation and ≃1% mobilisation frequencies (Figure 8). In both pNP40‐ and pUC11B‐mediated (co‐)mobilisation, in the absence of any *mobC* gene (pPEPi vector containing only the cognate *oriT* sequence), (co‐)mobilisation frequencies were comparable (*p* ≤ 0.001) to those of the pPEPi constructs containing the cognate *oriT* sequence and the *mobC* gene from either pUC11C (for pNP40) or pDRC3A (for pUC11C), suggesting that this *mobC* caused no significant effect in the (co‐)mobilisation of these constructs, and the existence of a specificity to each conjugative system. Finally, in the absence of nisin, no significant differences in (co‐)mobilisation compared to the empty vector‐harbouring strains were observed, highlighting the role of *mobC* expression in this process.

## DISCUSSION

The lactococcal conjugative plasmids pNP40 and pUC11B have been extensively characterised providing a significant understanding of the conjugation process and its regulation. It has previously been reported that pNP40 has the potential to mediate mobilisation of two smaller non‐conjugative plasmids present in the same donor strain (Ortiz Charneco et al., 2021). Not much was known about the mechanism of this mobilisation phenomenon prompting an analysis of the genetic determinants that promote plasmid mobilisation by pUC11B or pNP40. The ability to transfer mobilisable plasmids in addition to conjugative plasmids constitutes an outstanding scientific topic that could help us better understand how horizontal gene transfer processes shape bacterial populations. Moreover, this phenomenon is expected to become substantially relevant to the fermented food industry as it will offer opportunities to enhance the robustness and technological properties of lactococcal strains through conjugation‐mediated plasmid transfer events.

We report for the first time pUC11B‐mediated plasmid transfer of four out of the five other plasmids present in the same *L. lactis* UC11 strain following conjugation with recipient strain *L. cremoris* MG1614. This phenomenon is similar to that observed during a pNP40‐mediated conjugation event against the same recipient strain (Ortiz Charneco et al., 2021). The high co‐mobilisation frequencies observed prompted us to analyse the genetic determinants responsible for this co‐mobilisation phenotype presented by both pNP40 and pUC11B. It has been observed that mobilisable plasmids among *Staphylococcus aureus* strains (i.e., those lacking any conjugation‐related genes that are however mobilised by another, conjugative plasmid) require an *oriT* sequence similar to that present on the associated conjugative plasmid, and not *mob* genes as previously suggested (Ramsay & Firth, 2017). Our results agree with these reports, as no mobilisation‐related genes were observed in four out of the five mobilisable plasmids. Moreover, a previous study observed how the conjugative plasmid pLS20 from *Bacillus subtilis* is capable of mobilising other non‐conjugative elements containing its *oriT* sequence, even when this sequence was removed from pLS20 itself, showing how the *oriT* function of the conjugative plasmid is not itself required for the observed mobilisation phenomenon (Miyano et al., 2018).

Presumed *oriT* regions of both pNP40 and pUC11B were identified, with both sequences displaying a predicted nick region and an IR close to the relevant relaxase‐encoding gene, and a GC content of 24% and 32%, respectively. An interesting discovery was that the nick region from plasmid pUC11B is almost identical (except for the first four bases) to that predicted for plasmid pMRC01 (Hickey et al., 2001). Although the precise *nic*‐site of pUC11B and pNP40 remains to be elucidated, the work described herein signifies a substantial advance in current knowledge on how these conjugative plasmids mediate mobilisation of other non‐conjugative plasmids. After experimental determination of the pNP40 and pUC11B *oriT* sequences, the minimal sequences required for the mobilisation of the pUC11D, pUC11E, pUC11F, pDRC3E and pDRC3F by their respective conjugative plasmids were determined. Somewhat to our surprise, no obvious similarities exist between the *oriT* sequence of plasmid pNP40 and the minimal regions required for mobilisation of either pDRC3E or pDRC3F. However, the minimal sequences required for mobilisation of plasmids pUC11D, pUC11E and pUC11F are similar, though at varying levels, to the *oriT* sequence of plasmid pUC11B (Figure S3).

Plasmid pUC11A possesses the same *oriT* sequence as pUC11B and several *mob* genes, yet was not shown to be subject to mobilisation under the conditions tested. Plasmid pUC11A is a relatively large plasmid (59.284 kb), and its size may represent a limiting factor in the mobilisation ability of a plasmid. Perhaps mobilisation of plasmid pUC11A does occur, yet at such low frequencies that it was not observed under the conditions of the assay. A similar finding has been reported in *S. aureus*, in which the prevalence of conjugative plasmids is limited, although the presence of *oriT* sequences from the pWBG749‐ and pSK41‐family plasmids on numerous non‐conjugative plasmids facilitates the transfer of the latter (Ramsay et al., 2016). Relatively small plasmids are present among *S. aureus* strains when compared to other bacteria, suggesting that plasmid size may be a limiting factor for mobilisation, and since conjugation gene clusters are significantly bigger in size than mobilisation‐related loci, the presence of mobilisation loci among *S. aureus* plasmids may provide an evolutionary advantage to smaller non‐conjugative plasmids, enabling their presence among many strains while still preserving their potential mobilisation in the presence of a conjugative plasmid (Fernández‐López et al., 2014; Ramsay et al., 2016; Shearer et al., 2011). We hypothesise that the presence of an *oriT*‐like sequence in the smaller, non‐conjugative and mobilisable plasmids from *L. lactis* UC11 (and from *L. lactis* DRC3, despite a lack of similarity to *oriT*) signifies a relatively small impact on plasmid size when compared to the carriage of a full set of *mob* genes, and these sequences are likely promoting the high (co‐)mobilisation frequencies reported here. Moreover, small plasmids from *L. lactis*/*cremoris* strains replicate in a different manner from large plasmids (rolling circle replication as opposed to theta type replication, respectively) (Kelleher et al., 2019) and this may also affect the conjugation process, and therefore plasmid mobilisation, since conjugation and replication are mechanisms that promote plasmid dissemination (either horizontally or vertically, respectively) and that are connected to each other (Guzmán‐Herrador & Llosa, 2019).

Another interesting phenomenon observed in the (co‐)mobilisation assays was that mobilisation frequencies were significantly higher than the co‐mobilisation for all pNP40‐ and pUC11B‐mediated mobilisable plasmids, denoting that the reported smaller plasmids are capable of hijacking the conjugation machinery created by the conjugative plasmid and transfer to a recipient cell even when the conjugative plasmid itself is not transferred, a phenomenon that has previously been reported for the conjugative plasmid pMRC01 (Hickey et al., 2001). However, (co‐)mobilisation frequencies were significantly lower than the ones observed in the original donor strains, and our data indicate that this was due to the presence of *mobC* genes in the original hosts of the conjugative plasmids. There are two distinct versions of these genes that appear to promote mobilisation of either of the mobilisable plasmids from *L. lactis* UC11 or DRC3, and we hypothesise that MobC‐like proteins act as guides of the relaxase by binding to the *oriT* region and separating the strands, thus facilitating nicking of the DNA by the relaxase (Godziszewska et al., 2016; Zhang & Meyer, 1997).

However, it is important to note that plasmid size may be a limiting factor for high‐frequency mobilisation of non‐conjugative plasmids, i.e. large plasmids may mobilise at very low frequencies, or not be capable of mobilisation at all. The elucidation of the genetic determinants that promote high plasmid mobilisation provides mechanistic insights into how mobilisation occurs in *L. cremoris* and *L. lactis*, while it also represents a foundation for future studies on this phenomenon. Determination of the specific molecular mechanisms that underpin plasmid mobilisation could remove the plasmid size limitation, while also providing researchers with an invaluable tool to reliably enhance strain robustness in the dairy industry.

## AUTHOR CONTRIBUTIONS

**Guillermo Ortiz Charneco:** Conceptualization (equal); data curation (equal); formal analysis (lead); investigation (lead); validation (equal); writing – original draft (lead); writing – review and editing (equal). **Brian McDonnell:** Conceptualization (equal); data curation (equal). **Philip Kelleher:** Data curation (equal). **Andrius Buivydas:** Investigation (supporting). **Sofia Dashko:** Writing – review and editing (equal). **Paul P. de Waal:** Writing – review and editing (equal). **Irma van Rijswijck:** Writing – review and editing (equal). **Noël N. M. E. van Peij:** Writing – review and editing (equal). **Jennifer Mahony:** Conceptualization (equal); funding acquisition (equal); supervision (equal); validation (equal); writing – review and editing (equal). **Douwe Van Sinderen:** Conceptualization (equal); funding acquisition (equal); supervision (equal); validation (equal); writing – review and editing (equal).

## FUNDING INFORMATION

No funding information provided.

## CONFLICT OF INTEREST STATEMENT

SD, PdW, IvR and NvP are employed by the company dsm‐firmenich. The remaining authors declare that the research was conducted in the absence of any commercial or financial relationships that could be construed as a potential conflict of interest.

## Supporting information


Figure S1

Figure S2

Figure S3

Table S1

Table S2

Table S3

Table S4

Table S5
