# Supplementary figures and images for: Plasmid‐mediated horizontal gene mobilisation: Insights from two lactococcal conjugative plasmids
Source: Microb Biotechnol. 2024 May 16;17(5):e14421. doi: 10.1111/1751-7915.14421 (PMC11097999; doi:10.1111/1751-7915.14421)

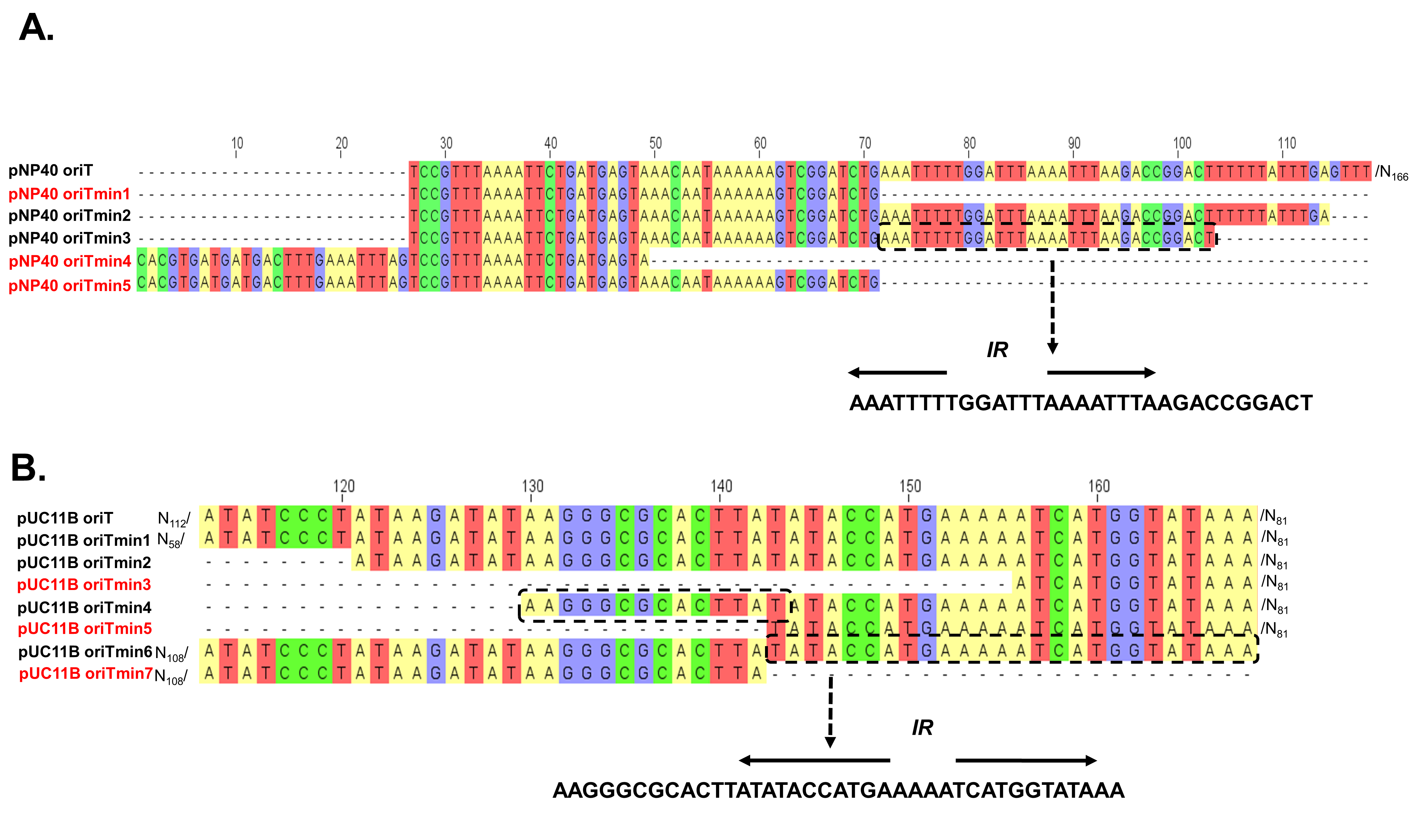

Supplement: Supplementary file 1 — Figure S1 [file MBT2-17-e14421-s004.tif]

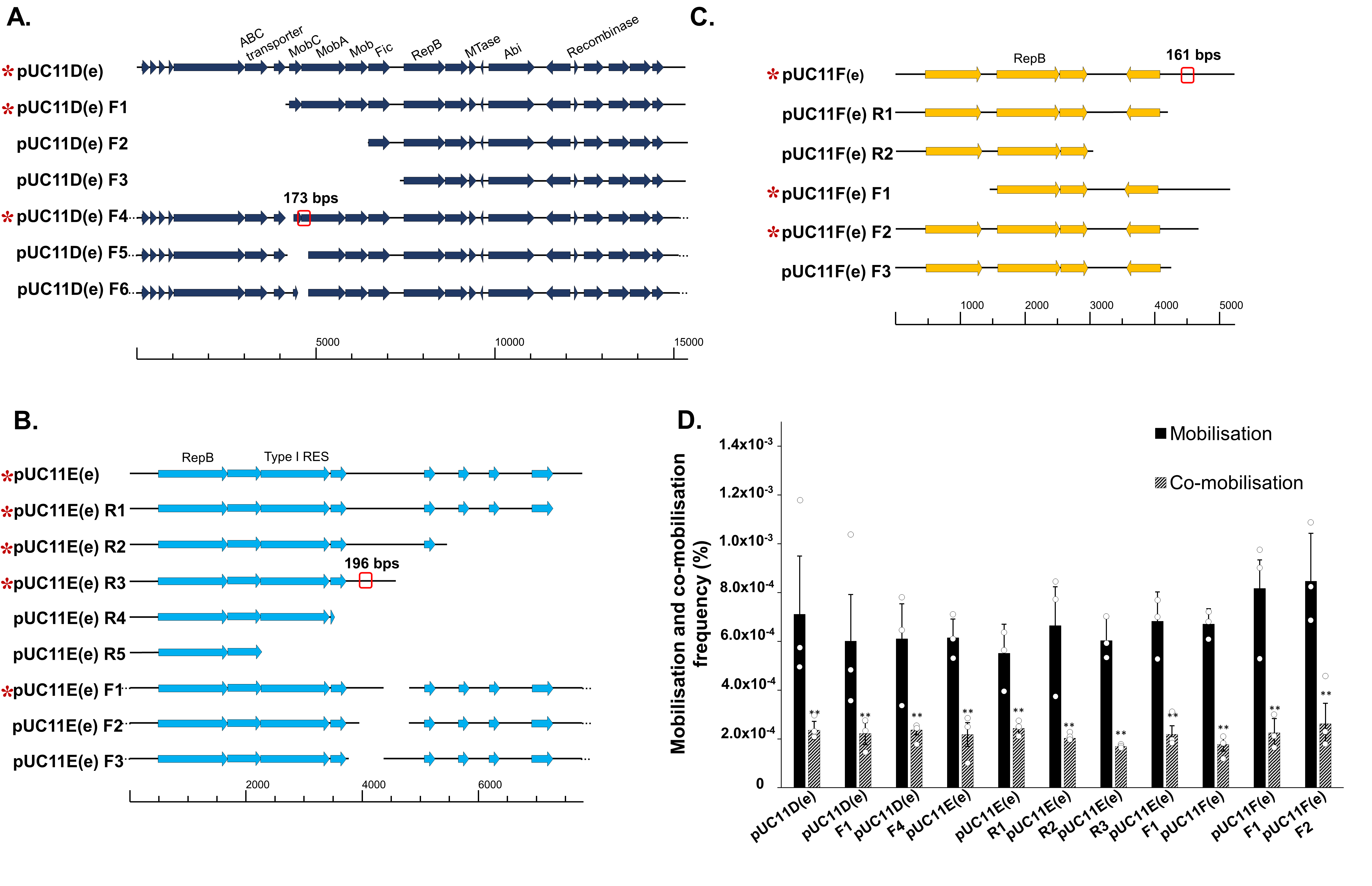

Supplement: Supplementary file 2 — Figure S2 [file MBT2-17-e14421-s008.tif]

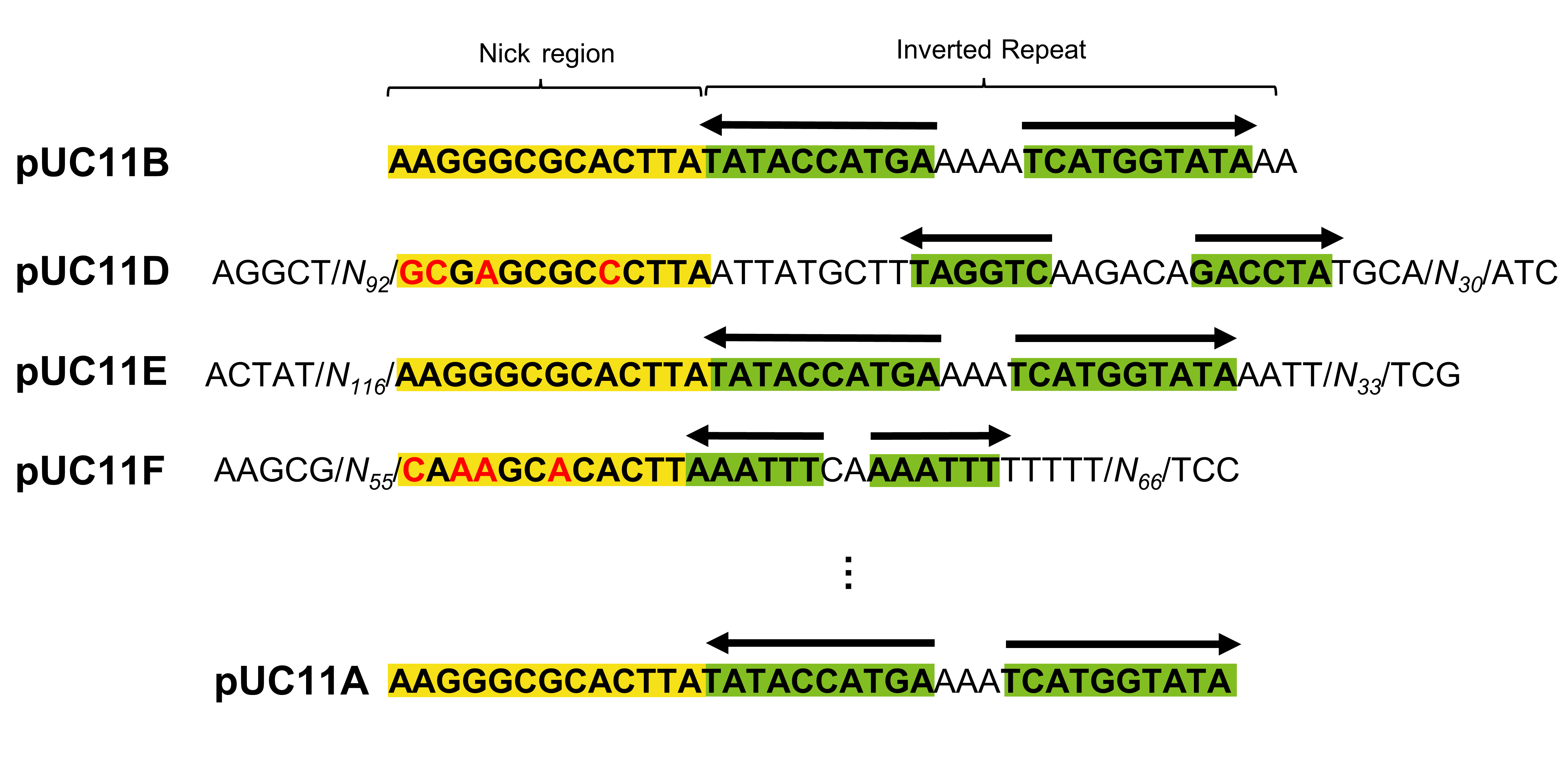

Supplement: Supplementary file 3 — Figure S3 [file MBT2-17-e14421-s007.tif]
