# Supplementary material for: Plasmid‐mediated horizontal gene mobilisation: Insights from two lactococcal conjugative plasmids
Source: Microb Biotechnol. 2024 May 16;17(5):e14421. doi: 10.1111/1751-7915.14421 (PMC11097999; doi:10.1111/1751-7915.14421)
Supplement: Supplementary file 4 — Table S1 [file MBT2-17-e14421-s002.docx]

**Supplementary Table S1.** Lactococcal strains used in this study.

| **Strain** | **Plasmids present in the strain** | **Relevant properties** |
| --- | --- | --- |
| **L. lactis UC11** | pUC11A, pUC11B, pUC11C, pUC11D, pUC11E, pUC11F | Donor strain, harbouring the conjugative plasmid pUC11B, conferring tetracycline resistance, and the (co-)mobilisable plasmids pUC11D, pUC11E and pUC11F |
| **L. lactis DRC3** | pDRC3A, pDRC3B(pNP40), pDRC3C, pDRC3D, pDRC3E, pDRC3F, pDRC3G | Strain harbouring the conjugative plasmid pNP40, conferring nisin resistance, and the (co-)mobilisable plasmid pDRC3E and pDRC3F |
| **L. cremoris MG1614** | - | Main recipient strain, plasmid-free and streptomycin resistant |
| **L. cremoris NZ9000** | - | Derivative of the parental strain *L. cremoris* MG1363 |
| **L. cremoris NZ9000** | pNP40 | Strain harboring the conjugative plasmid pNP40, resistant to nisin |
| **L. cremoris NZ9000** | pUC11B | Strain harboring the conjugative plasmid pUC11B, resistant to tetracycline |
