# Supplementary material for: Plasmid‐mediated horizontal gene mobilisation: Insights from two lactococcal conjugative plasmids
Source: Microb Biotechnol. 2024 May 16;17(5):e14421. doi: 10.1111/1751-7915.14421 (PMC11097999; doi:10.1111/1751-7915.14421)
Supplement: Supplementary file 5 — Table S2 [file MBT2-17-e14421-s003.docx]

**Supplementary Table S2.** Oligonucleotides used in this study.

| Oligonucleotide name | Oligonucleotide sequence (5’ → 3’) |
| --- | --- |

| Co-mobilisation screening primers |  |
| --- | --- |
| ScrnUC11-Fw | GAGCCTTCGGTAGAAGAC |
| ScrnUC11-Rv | CAACATGACGAATTCCTAAAC |
| ScrnUC11A-Fw | GTTTTGGGATTTATTCTATTAGGA |
| ScrnUC11A-Rv | CCAACAATGATTTCTTCTGAAAC |
| ScrnUC11B-Fw | CGGTATCTTAGCACATGTTG |
| ScrnUC11B-Rv | CATGGTTCAATTGTTGTTTGTAA |
| ScrnUC11C-Fw | GAAGGCTGGACGATAACC |
| ScrnUC11C-Rv | CTGCCATCTTGACTTCCC |
| ScrnUC11D-Fw | CTAAGACTGGACTACAAAAACA |
| ScrnUC11D-Rv | GCAGCTGAATTAGGGTTATC |
| ScrnUC11E-Fw | GCGGATGAGTTAGGTGTG |
| ScrnUC11E-Rv | CTTTAAGAGCGTTCACTTCAT |
| ScrnUC11F-Fw | GCTAGTCAAACGAACAGTAAG |
| ScrnUC11F-Rv | CAGGAGTCTTTGAAATGTCTAA |
|  |  |
| Minimal co-mobilisation primers^ᴪ^ |  |
| Erm_1-Fw | AAAAAAGAGCTCACCAAGACGAAGAG |
| Erm_1-Rv | AAAAAAGTCGACGACATACTGTTCTTCC |
| Erm_2-Fw | AAAAAAGTCGACACCAAGACGAAGAG |
| Erm_2-Rv | AAAAAACCATGGGACATACTGTTCTTCC |
| Erm_3-Fw | AAAAAAGAGCTCACCAAGACGAAGAG |
| Erm_3-Rv | AAAAAACCATGGGACATACTGTTCTTCC |
| pDRC3E_Full-Fw | AAAAAAGTCGACGTGTAAGTGCGCATTGCA |
| pDRC3E_Full-Rv | AAAAAAGAGCTCGATGTCGTGCTAACCCCTA |
| pDRC3E_R1-Rv | AAAAAAGAGCTCCCATGGATGAACAAACAAAAA |
| pDRC3E_R2-Rv | AAAAAAGAGCTCTTATCACTCCTCTTTTAACTTAAT |
| pDRC3E_R3-Rv | AAAAAAGAGCTCGGTTAGATCTTAGGGGCTAT |
| pDRC3E_R4-Rv | AAAAAAGAGCTCGCAAGCTCCTTAATCGTTTTT |
| pDRC3E_F1-Fw | AAAAAAGTCGACGTGTTTTCAAGGGTATTAAGATT |
| pDRC3E_F2-Fw | AAAAAAGTCGACCTGAGGCATTTTATCTTTGAG |
| pDRC3E_F2-Rv | AAAAAAGAGCTCATTCGGTTACAATCCTCACA |
| pDRC3E_F3-Rv | AAAAAAGAGCTCCACTTTCCCCTTTTATCCC |
| pDRC3F_Full-Fw | AAAAAACCATGGCGTCACGGAGTGTTTCTG |
| pDRC3F_Full-Rv | AAAAAAGAGCTCCGTAGAAGTCTGACAGAGTT |
| pDRC3F_F1-Fw | AAAAAACCATGGCTTCATTGATACCAGTTTTAAAG |
| pDRC3F_R1-Rv | AAAAAAGAGCTCACATAAAAACAACCCCTATTACC |
| pDRC3F_R2-Rv | AAAAAAGAGCTCCTTGGTTTTTCTTGTTCTTCTAT |
| pDRC3F_F2-Fw | AAAAAACCATGGCGTGCTAGATTACTTGGTTCA |
| pDRC3F_F2-Rv | AAAAAAGAGCTCCCAAGCGGGTAAACTTATAAG |
| pDRC3F_F3-Rv | AAAAAAGAGCTCCCGCCCCATTAAGTAGAGT |
| pUC11D_Full-Fw | AAAAAAGTCGACTGAAAAGAAAAAAGACCTATGG |
| pUC11D_Full-Rv | AAAAAAGAGCTCAAACGCACCTCCATTATTTATAA |
| pUC11D_F1-Fw | AAAAAAGTCGACGACAAAGGGCGATTGAGGA |
| pUC11D_F2-Fw | AAAAAAGTCGACGTGAGGGGATATTTTGGTTTT |
| pUC11D_F3-Fw | AAAAAAGTCGACCTATCCCAGAAAAAACTTGTTTA |
| pUC11D_F4-Fw | AAAAAAGTCGACCGACAACTTCATAACCAAACC |
| pUC11D_F4-Rv | AAAAAAGAGCTCGGCGATAAAGGGATCAATCC |
| pUC11D_F5-Rv | AAAAAAGAGCTCCGATCAGCTCTTTATCTTGG |
| pUC11D_F6-Fw | AAAAAAGTCGACCAATTGGAACGTCCTGTGG |
| pUC11E_Full-Fw | AAAAAACCATGGATAAAGATCAGCAAGTTCTATCA |
| pUC11E_Full-Rv | AAAAAAGTCGACCGCTGGATAGCATAATGATTAT |
| pUC11E_R1-Rv | AAAAAAGTCGACGTCTAAACGCTTTGAATAGTC |
| pUC11E_R2-Rv | AAAAAAGTCGACGGGTCTTAGGTACTCCTAAA |
| pUC11E_R3-Rv | AAAAAAGTCGACCTAAACCCTAAAAACCTGTATC |
| pUC11E_R4-Rv | AAAAAAGTCGACCATCTAGTAAAATTTGGTTGTTCT |
| pUC11E_R5-Rv | AAAAAAGTCGACGCGTTCACTTCATCTTTATATTG |
| pUC11E_F1-Fw | AAAAAACCATGGGCCCCAATCCCCATATTCT |
| pUC11E_F1-Rv | AAAAAAGTCGACTTTACATGAGCAAAGCGAGTT |
| pUC11E_F2-Rv | AAAAAAGAGCTCCGATCAGCTCTTTATCTTGG |
| pUC11E_F3-Fw | AAAAAAGTCGACCAATTGGAACGTCCTGTGG |
| pUC11F_Full-Fw | AAAAAAGTCGACGGAGCGACAAGATCATTCA |
| pUC11F_Full-Rv | AAAAAAGAGCTCGCTTGTACACCTCGATAAAAG |
| pUC11F_R1-Rv | AAAAAAGAGCTCCACTGTTCTCACTATCTTTTTTT |
| pUC11F_R2-Rv | AAAAAAGAGCTCGGCTTCGGATGAACTTTCTT |
| pUC11F_F1-Rv | AAAAAAGTCGACGCTCCTCTTTTTGTTATAATGTT |
| pUC11F_F2-Fw | AAAAAACCATGGGTTGCTTGCCAGTCTTTTTTA |
| pUC11F_F2-Rv | AAAAAAGAGCTCGGAGTACCTAAGACCCTTT |
| pUC11F_F3-Rv | AAAAAAGAGCTCCCGCTCGCAGATACTTTG |
|  |  |
| Minimal oriT primers^ᴪ^ |  |
| pNP40_oriT-Fw | AAAAAACCATGGTCCGTTTAAAATTCTGATGAGTA |
| pNP40_oriT-Rv | AAAAAAGAGCTCAACGCCATCAGAATGCTTTTA |
| pNP40_oriTmin1-Fw | AAAAAACCATGGTCCGTTTAAAATTCTGATGAGTA |
| pNP40_oriTmin1-Rv | AAAAAAGAGCTCCAGATCCGACTTTTTTATTG |
| pNP40_oriTmin2-Rv | AAAAAAGAGCTCTCAAATAAAAAAGTCCGGTCTTA |
| pNP40_oriTmin3-Rv | AAAAAAGAGCTCAGTCCGGTCTTAAATTTTAAATC |
| pNP40_oriTmin4-Fw | AAAAAACCATGGCACGTGATGATGACTTTGAAAT |
| pNP40_oriTmin4-Rv | AAAAAAGAGCTCTACTCATCAGAATTTTAAACGG |
| pNP40_oriTmin5-Rv | AAAAAAGAGCTCCAGATCCGACTTTTTTATTGTT |
| pUC11B_oriT-Fw | AAAAAACCATGGAACTTCCTAGTCCTTTCTTATTT |
| pUC11B_oriT-Rv | AAAAAAGAGCTCAAGAAGTGTTCTCCTTTCTTTTT |
| pUC11B_oriTmin1-Fw | AAAAAACCATGGATGCAACAAAATAAGGCAAAAGA |
| pUC11B_oriTmin1-Rv | AAAAAAGAGCTCAAGAAGTGTTCTCCTTTCTTTTT |
| pUC11B_oriTmin2-Fw | AAAAAACCATGGATAAGATATAAGGGCGCACTT |
| pUC11B_oriTmin3-Fw | AAAAAACCATGGATCATGGTATAAATTGCCAATCT |
| pUC11B_oriTmin4-Fw | AAAAAACCATGGAAGGGCGCACTTATATACC |
| pUC11B_oriTmin4-Rv | AAAAAAGAGCTCAAGAAGTGTTCTCCTTTCTTTTT |
| pUC11B_oriTmin5-Fw | AAAAAACCATGGTATACCATGAAAAATCATGGT |
| pUC11B_oriTmin6-Fw | AAAAAACCATGGTCCTAGTCCTTTCTTATTTGA |
| pUC11B_oriTmin6-Rv | AAAAAAGAGCTCTTTATACCATGATTTTTCATGGT |
| pUC11B_oriTmin7-Rv | AAAAAAGAGCTCTAAGTGCGCCCTTATATCTTAT |
|  |  |
| Mob genes cloning primers^ᴪ^ |  |
| pDRC3A_mobC-Fw | AGCAGCGGATCCAGGAGGCACTCACCATGAAAAAAACCAAAAATCGTGAG |
| pDRC3A_mobC-Rv | AGCAGCGAATTCTTAGCCCTCCGAAATATATTG |
| pDRC3A_Tandem-Rv | AGCAGCGAATTCCGGACTAAATTTCAAAATCATCA |
| pUC11A_mobC-Fw | AGCAGCGGATCCAGGAGGCACTCACCATGAAAAAAACCAAAAATCGTGAG |
| pUC11A_mobC-Rv | AGCAGCGAATTCCTAGCCCTCCGAAATAAAA |
| pUC11A_Tandem-Rv | AGCAGCGAATTCTCAATCATTAGGACGGTCA |
| pUC11C_mobC-Fw | AGCAGCGGATCCAGGAGGCTATTTATGGATCAGA |
| pUC11C_mobC-Rv | AGCAGCGAATTCGCTTATTTTTGCAATTGTTGCC |
| pUC11C_Tandem-Rv | AGCAGCGAATTCCTATTTTTTCGGAAAGATTCTTTT |
| pDRC3A_mobA-Fw | AGCAGCGGATCCAGGAGGCACTCACCATGACAGTAATTTATATGCCTAAA |
| pDRC3A_mobA-Rv | AGCAGCGAATTCCGGACTAAATTTCAAAATCATCA |
| pUC11C_mobA-Fw | AGCAGCGGATCCAGGAGGCACTCACCATGGCAACAATTGCAAAAATAAG |
| pUC11C_mobA-Rv | AGCAGCGAATTCCTATTTTTTCGGAAAGATTCTTTT |
|  |  |
| oriT cloning into pPEPi^ᴪ^ |  |
| pNP40_oriT_pPTPi-Fw | AAAAAACCCGGGTCCGTTTAAAATTCTGATGAGTA |
| pNP40_oriT_pPTPi-Rv | AAAAAAGCATGCAGTCCGGTCTTAAATTTTAAATC |
| pUC11B_oriT_pPTPi-Fw | AAAAAACCCGGGAGTGGTAAAATATCCCTATAAGA |
| pUC11B_oriT_pPTPi-Rv | AAAAAAGCATGCGAAGTGTTCTCCTTTCTTTTTT |
|  |  |
| pNZ8048E assembly^ᴪ^ |  |
| pNZ8048-Fw | AAAAAATGTACATATGAGATAATGCCGACTGTACT |
| pNZ8048-Rv | AAAAAAGAATTCTTGATATGCCTCCTAAATTTTTATC |
| Ery-Fw | AAAAAAGAATTCACCAAGACGAAGAG |
| Ery-Rv | AAAAAATGTACAGACATACTGTTCTTCC |

ᴪ Introduced restriction enzyme sites are single underlined, whereas, Shine-Dalgarno sequences from pNZ8048 are doubly underlined.

₳ Hexa His Tag sequences required for Ni-NTA purification are doubly underlined
