# Supplementary material for: Plasmid‐mediated horizontal gene mobilisation: Insights from two lactococcal conjugative plasmids
Source: Microb Biotechnol. 2024 May 16;17(5):e14421. doi: 10.1111/1751-7915.14421 (PMC11097999; doi:10.1111/1751-7915.14421)
Supplement: Supplementary file 6 — Table S3 [file MBT2-17-e14421-s006.docx]

**Supplementary Table S3.** Plasmids and plasmid constructs employed in this study.

| **Plasmid** | **Characteristics** | **Plasmid size (bps)** | **Reference** |
| --- | --- | --- | --- |
| **pNZ44E** | Erythromycin-resistant, *E,coli*-*L, lactis* shuttle vector pNZ44, with a P44 constitutive promoter | 3,394 | Draper *et al*,. 2009 |
| **pNZ8048E** | Erythromycin-resistant version of the high-copy-number *E, coli*-*L, lactis* overexpression vector, P*nisA;* Erm^r^ | 3,349 | This study |
| **pNZ8048E::** **oriT_pNP40_** | pNZ8048E derivative harbouring the pNP40 *oriT*-containing region | 3,614 | This study |
| **pNZ8048E::** **oriTmin1-5_pNP40_** | pNZ8048E derivatives containing one of the five fragments from the pNP40 *oriT*-containing region | 3,394-3,437-3,426-3,398-3,420 | This study |
| **pNZ8048E::oriT_pUC11B_** | pNZ8048E derivative harbouring the pUC11B *oriT*-containing region | 3,598 | This study |
| **pNZ8048E::oriTmin1-7_pUC11B_** | pNZ8048E derivatives containing one of the seven fragments from the pUC11B *oriT*-containing region | 3,544-3,478-3,443-3,469-3,457-3,513-3,487 | This study |
| **pPTPi** | Low-copy-number *E, coli-L, lactis* vector, Tc^r^, P*nisA*, pPTP derivative | 6,837 | O’Driscoll *et al*,. 2004 |
| **pPEPi** | Low-copy-number *E, coli-L, lactis* vector, Erm^r^, P*nisA*, pPTPi derivative | 6,916 | Ortiz Charneco *et al*,. 2023 |
| **pPTPi::mob** | Six pPTPi constructs containing a copy of *mobA* or *mobC* from either pUC11C or pDRC3A, or a copy of both *mobA* and *mobC* (in tandem) from these plasmids | 8,690-7,217-8,359-7,206-9,046-8,732- | This study |
| **pPEPi::mob::oriT_pNP40_** | Four pPEPi constructs containing a copy of *mobC* from either pUC11C or pDRC3A, or a copy of both *mobC* and *mobA* (in tandem) from these plasmids, as well as the *oriT* sequence from pNP40 | 7,373-7,362-9,202-8,888 | This study |
| **pPEPi::mob::oriT_pUC11B_** | Four pPEPi constructs containing a copy of *mobC* from either pUC11C or pDRC3A, or a copy of both *mobC* and *mobA* (in tandem) from these plasmids, as well as the *oriT* sequence from pUC11B | 7,440-7,429-9,269-8,955 | This study |
| **pDRC3E(e)** | Erythromycin derivative of plasmid pDRC3E | 5,813 | This study |
| **pDRC3E(e) R1-R4, F1-F3** | Erythromycin derivatives of plasmid pDRC3E, of seven different sizes | 5,310-4,542-3,884-2,026-5,229-5,771-5,649 | This study |
| **pDRC3F(e)** | Erythromycin derivative of plasmid pDRC3F | 3,705 | This study |
| **pDRC3F(e) R1-R2, F1-F3** | Erythromycin derivatives of plasmid pDRC3F, of five different sizes | 2,994-1,777-3,434-3,657-3,468 | This study |
| **pUC11D(e)** | Erythromycin derivative of plasmid pUC11D | 15,393 | This study |
| **pUC11D(e) F1-F6** | Erythromycin derivatives of plasmid pUC11D, of six different sizes | 11,196-8,937-8,057-15,372-15,119-15,221 | This study |
| **pUC11E(e)** | Erythromycin derivative of plasmid pUC11E | 7,809 | This study |
| **pUC11E(e) R1-R5, F1-F3** | Erythromycin derivatives of plasmid pUC11E, of eight different sizes | 6,881-5,416-4,495-3,489-2,166-7,663-7,452-7,614 | This study |
| **pUC11F(e)** | Erythromycin derivative of plasmid pUC11F | 5,238 | This study |
| **pUC11F(e) R1-R2, F1-F3** | Erythromycin derivatives of plasmid pUC11F, of five different sizes | 4,191-3,175-3,775-5,188-5,028 | This study |
