# Supplementary material for: Plasmid‐mediated horizontal gene mobilisation: Insights from two lactococcal conjugative plasmids
Source: Microb Biotechnol. 2024 May 16;17(5):e14421. doi: 10.1111/1751-7915.14421 (PMC11097999; doi:10.1111/1751-7915.14421)
Supplement: Supplementary file 7 — Table S4 [file MBT2-17-e14421-s001.docx]

**Supplementary Table S4.** Locations and sizes of the different and subsequently smaller fragments of the *oriT*-containing sequences of the conjugative plasmids pNP40 and pUC11B, which were all individually cloned into the expression vector pNZ8048E. Fragments positions are either given in relation to the last nucleotide of the relaxase-encoding gene from plasmid pNP40, *traA_b_*, or in relation to the first nucleotide of the relaxase-encoding gene *trsA* from plasmid pUC11B. (Co-)mobilisation of each pNZ8048E construct is depicted by a ‘+’, whereas no visible co-mobilisation is depicted by a ‘-’.

| Fragments’ name | Fragments’ Start position | Fragments’ End position | Fragments’ length (bp) | (Co-)mobilisation observed (+/-) |
| --- | --- | --- | --- | --- |
| *oriT*_pNP40_ | +1 | +265 | 265 | + |
| *oriT*min1_pNP40_ | +1 | +45 | 45 | - |
| *oriT*min2_pNP40_ | +1 | +88 | 88 | + |
| *oriT*min3_pNP40_ | +1 | +77 | 77 | + |
| *oriT*min4_pNP40_ | -25 | +23 | 49 | - |
| *oriT*min5_pNP40_ | -25 | +45 | 71 | - |
| *oriT*_pUC11B_ | -249 | -1 | 249 | + |
| *oriT*min1_pUC11B_ | -195 | -1 | 195 | + |
| *oriT*min2_pUC11B_ | -129 | -1 | 129 | + |
| *oriT*min3_pUC11B_ | -94 | -1 | 94 | - |
| *oriT*min4_pUC11B_ | -120 | -1 | 120 | + |
| *oriT*min5_pUC11B_ | -107 | -1 | 108 | - |
| *oriT*min6_pUC11B_ | -245 | -82 | 164 | + |
| *oriT*min7_pUC11B_ | -245 | -108 | 138 | - |
