# Supplementary material for: Plasmid‐mediated horizontal gene mobilisation: Insights from two lactococcal conjugative plasmids
Source: Microb Biotechnol. 2024 May 16;17(5):e14421. doi: 10.1111/1751-7915.14421 (PMC11097999; doi:10.1111/1751-7915.14421)
Supplement: Supplementary file 8 — Table S5 [file MBT2-17-e14421-s005.docx]

**Supplementary Table S5.** Locations and sizes of the different and subsequently smaller versions of each erythromycin derivative of the mobilisable plasmids (pDRC3E, pDRC3F, pUC11D, pUC11E and pUC11F). Nucleotide start (forward and reverse primers) positions are given in relation to the plasmids’ replication gene. (Co-)mobilisation of each plasmid version is depicted by a ‘+’, whereas no visible co-mobilization is depicted by a ‘-’.

| Plasmid name | Start  (Forward) | Start  (Reverse) | Plasmid length (bp) | (Co-)mobilisation Observed (+/-) |
| --- | --- | --- | --- | --- |
| pDRC3E(e) | 5033 | 5020 | 5813 | + |
| pDRC3E(e) R1 | 5033 | 4517 | 5310 | - |
| pDRC3E(e) R2 | 5033 | 3749 | 4542 | - |
| pDRC3E(e) R3 | 5033 | 3091 | 3884 | - |
| pDRC3E(e) R4 | 5033 | 1233 | 2026 | - |
| pDRC3E(e) F1 | 5617 | 5020 | 5229 | + |
| pDRC3E(e) F2 | 4863 | 4808 | 5771 | + |
| pDRC3E(e) F3 | 4863 | 4686 | 5649 | - |
| pDRC3F(e) | 3142 | 3141 | 3705 | + |
| pDRC3F(e) R1 | 3142 | 2430 | 2994 | - |
| pDRC3F(e) R2 | 3142 | 1213 | 1777 | - |
| pDRC3F(e) F1 | 3413 | 3141 | 3434 | + |
| pDRC3F(e) F2 | 2899 | 2848 | 3657 | + |
| pDRC3F(e) F3 | 2899 | 2659 | 3468 | - |
| pUC11D(e) | 7937 | 7936 | 15393 | + |
| pUC11D(e) F1 | 12134 | 7936 | 11196 | + |
| pUC11D(e) F2 | 14393 | 7936 | 8937 | - |
| pUC11D(e) F3 | 15273 | 7936 | 8057 | - |
| pUC11D(e) F4 | 12751 | 12729 | 15372 | + |
| pUC11D(e) F5 | 12751 | 12476 | 15119 | - |
| pUC11D(e) F6 | 12649 | 12476 | 15221 | - |
| pUC11E(e) | 7321 | 7320 | 7809 | + |
| pUC11E(e) R1 | 7321 | 6392 | 6881 | + |
| pUC11E(e) R2 | 7321 | 4927 | 5416 | + |
| pUC11E(e) R3 | 7321 | 4006 | 4495 | + |
| pUC11E(e) R4 | 7321 | 3000 | 3489 | - |
| pUC11E(e) R5 | 7321 | 1677 | 2166 | - |
| pUC11E(e) F1 | 3936 | 3789 | 7663 | + |
| pUC11E(e) F2 | 3936 | 3578 | 7452 | - |
| pUC11E(e) F3 | 3774 | 3578 | 7614 | - |
| pUC11F(e) | 3676 | 3675 | 5238 | + |
| pUC11F(e) R1 | 3676 | 2628 | 4191 | - |
| pUC11F(e) R2 | 3676 | 1612 | 3175 | - |
| pUC11F(e) F1 | 5139 | 3675 | 3775 | + |
| pUC11F(e) F2 | 2991 | 3080 | 5188 | + |
| pUC11F(e) F3 | 2991 | 3239 | 5028 | - |
